# Role of c-Met/**β**1 integrin complex in the metastatic cascade in breast cancer

**DOI:** 10.1172/jci.insight.138928

**Published:** 2021-06-22

**Authors:** Darryl Lau, Harsh Wadhwa, Sweta Sudhir, Alexander Chih-Chieh Chang, Saket Jain, Ankush Chandra, Alan T. Nguyen, Jordan M. Spatz, Ananya Pappu, Sumedh S. Shah, Justin Cheng, Michael M. Safaee, Garima Yagnik, Arman Jahangiri, Manish K. Aghi

**Affiliations:** 1 Department of Neurological Surgery, UCSF, San Francisco, California, USA.; 2 Department of Neurological Surgery, Emory University, Atlanta, Georgia, USA.

**Keywords:** Oncology, Cancer, Integrins

## Abstract

Metastases cause 90% of human cancer deaths. The metastatic cascade involves local invasion, intravasation, extravasation, metastatic site colonization, and proliferation. Although individual mediators of these processes have been investigated, interactions between these mediators remain less well defined. We previously identified a complex between receptor tyrosine kinase c-Met and β1 integrin in metastases. Using cell culture and in vivo assays, we found that c-Met/β1 complex induction promoted intravasation and vessel wall adhesion in triple-negative breast cancer cells, but did not increase extravasation. These effects may have been driven by the ability of the c-Met/β1 complex to increase mesenchymal and stem cell characteristics. Multiplex transcriptomic analysis revealed upregulated Wnt and hedgehog pathways after c-Met/β1 complex induction. A β1 integrin point mutation that prevented binding to c-Met reduced intravasation. OS2966, a therapeutic antibody disrupting c-Met/β1 binding, decreased breast cancer cell invasion and mesenchymal gene expression. Bone-seeking breast cancer cells exhibited higher levels of c-Met/β1 complex than parental controls and preferentially adhered to tissue-specific matrix. Patient bone metastases demonstrated higher c-Met/β1 complex than brain metastases. Thus, the c-Met/β1 complex drove intravasation of triple-negative breast cancer cells and preferential affinity for bone-specific matrix. Pharmacological targeting of the complex may have prevented metastases, particularly osseous metastases.

## Graphical abstract

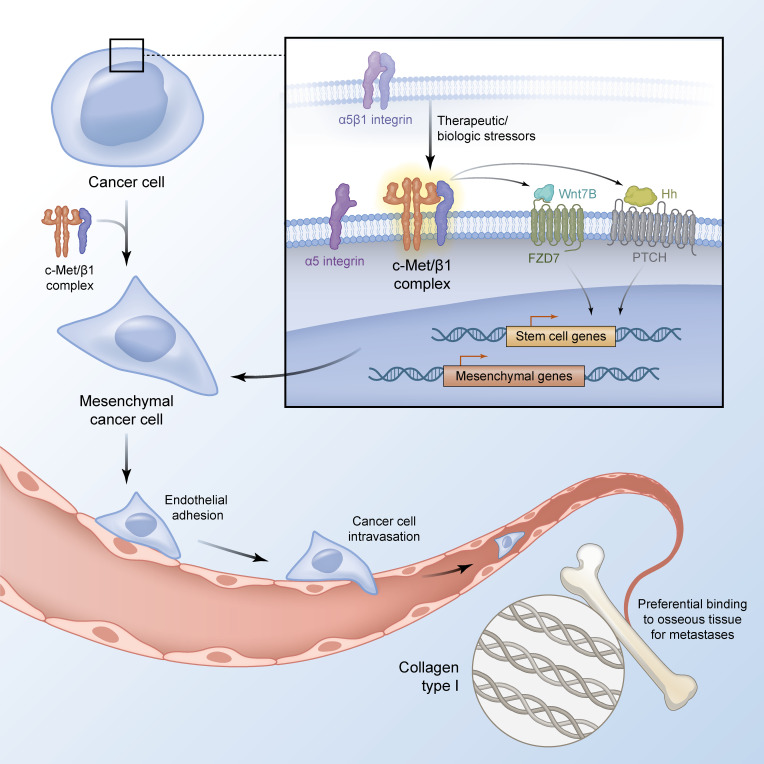

## Introduction

Metastatic spread of cancer, one of the hallmarks of malignancy, is the main cause of up to 90% of human cancer deaths (1) and confers a median survival of less than 6 months once identified (2). Despite the clinical importance and tremendous public health impact of metastases, there remains insufficient understanding of the underlying molecular mechanisms of metastases, and, importantly, this results in a lack of potential targetable vulnerabilities of the metastatic process.

The metastatic cascade comprises 5 major steps: local invasion of tumor cells at the primary site, transendothelial migration of tumor cells into the circulation (intravasation), egress out of the circulation and into sites of intended metastasis (extravasation), colonization at the metastatic site, and proliferation at the metastatic site leading to clinically detectable metastases (3). The role of specific mediators in early versus later steps of the metastatic cascade remains in need of clarification. The metastatic process is highly inefficient, as only 0.01% of cancer cells released into the circulation develop into metastatic foci (4), underscoring the importance of identifying the most vulnerable steps of the cascade and developing therapies targeting those steps.

There is evidence suggesting the importance of the latter steps in the cascade dating back to over a century ago, when Paget suggested that metastasis is not due to chance, but rather that some tumor cells (the “seed”) grow preferentially in the microenvironment of select organs (the “soil”) and that metastases result only from the appropriate seed in suitable soil (5). More modern gene expression studies have supported the hypothesis that tumor cells gain “metastasis virulence genes” that fail to affect primary tumor development and confer survival advantages only within the context of specific foreign microenvironments (6).

Although individual mediators of these processes have been investigated, the interactions between these mediators have been less well studied. We previously demonstrated an increased formation of a structural complex between receptor tyrosine kinase c-Met and β1 integrin in metastases compared with primary tumors (7). Here, we used cell culture models and in vivo assays to reveal the role of the c-Met/β1 complex in specific steps of the metastatic cascade in breast cancer.

The data presented in this study show that the c-Met/β1 complex drove breast cancer cell intravasation and induced preferential affinity for tissue-specific matrix, particularly for osseous sites. This phenotype is secondary to changes in mesenchymal gene expression profile and increased stem cell characteristics. These findings reveal that the c-Met/β1 complex may be a viable pharmacologic target to prevent metastases.

## Results

### The c-Met/β1 complex promoted expression of genes related to cancer pathways and progression in breast cancer cells.

**Figure 1:**
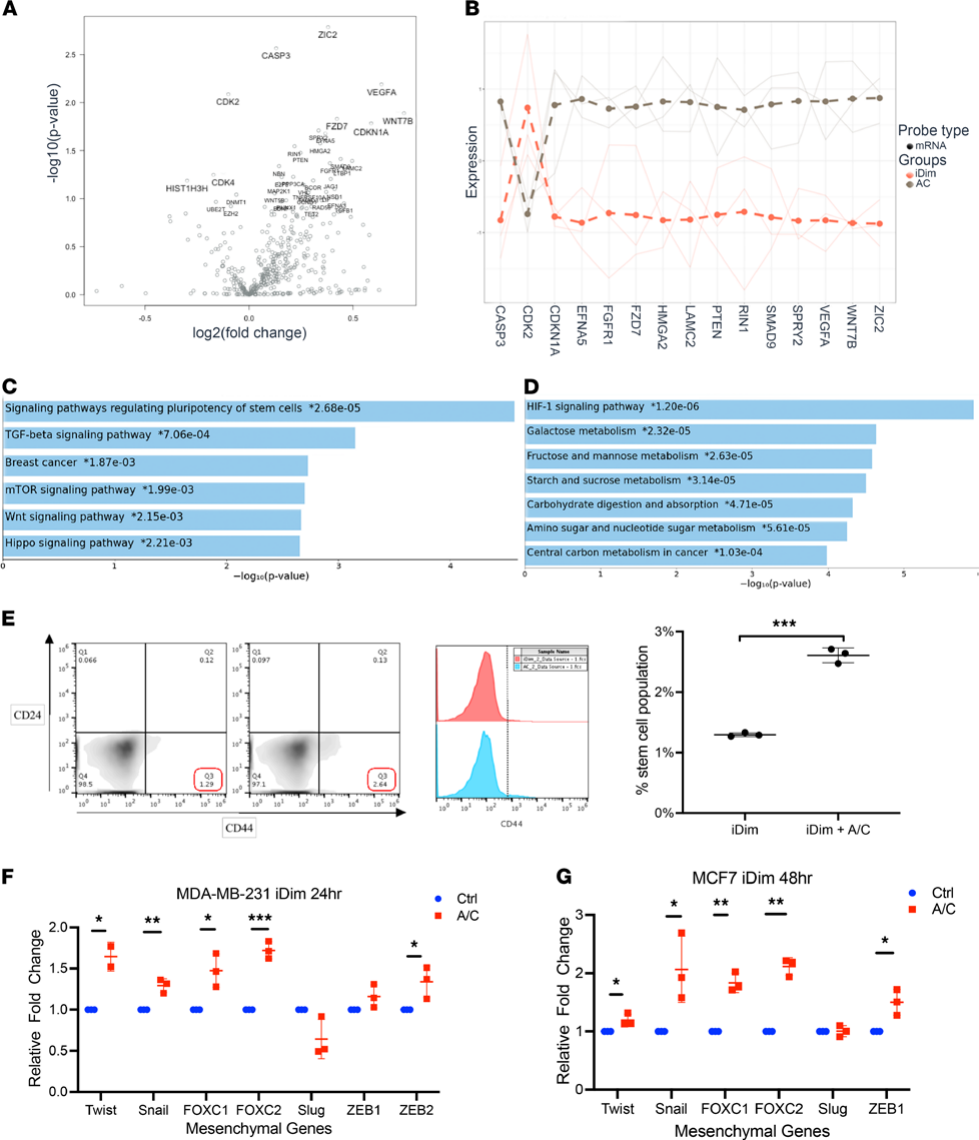
c-Met/β1 complex formation activates pathways implicated in metastases in breast cancer cells. Treatment of MDA-MB-231-iDimerize-c-Met-β1 cells with AP21967 (A/C ligand)–induced c-Met/β1 complex formation, leading to the upregulation of many of the 770 genes from 13 cancer-associated canonical pathways in the NanoString nCounter platform (*n* = 3/group), as evidenced by (**A**) volcano plot revealing the most upregulated genes with the largest fold change in the upper right; (**B**) top 15 hits based on *P* values; and (**C**) elevated expression of genes in the stem cell, TGF-β, mTOR, and Wnt signaling pathways (*n* = 3/group). (**D**) Multiplex transcriptomic analysis revealed that AP21967 upregulated the expression of genes in several downstream cancer progression pathways in MDA-MB-231-iDimerize-c-Met-β1, including increased HIF-1 signaling pathway and a variety of metabolic pathways (*n* = 3/group). (**E**) Flow cytometry revealed that AP21967 doubled the CD44^+^CD24^–^ stem cell fraction of MDA-MB-231-iDimerize-c-Met-β1 cells (*n* = 3/group; scatter dot plot with horizontal line at mean and vertical line representing SD; unpaired *t* test; *P* < 0.001). To determine whether c-Met/β1 complex formation enriched the expression of mesenchymal transcription factors, qRT-PCR was performed for 6 mesenchymal transcription factors (*Twist*, *Snail*, *FOXC1*, *FOXC2*, *Slug*, *ZEB1*, and *ZEB2*) in (**F**) MDA-MB-231-iDimerize-c-Met-β1 cells and (**G**) MCF7-iDimerize-c-Met-β1 cells treated with or without 0.5 nM AP21967 (A/C ligand) for 24 hours (*n* = 3/group; scatter dot plot with horizontal line at mean and vertical line representing SD; unpaired *t* test). Note that *ZEB2* was not detectable in MCF7-iDimerize-c-Met-β1 cells. *P < 0.05; **P < 0.01; ***P < 0.001.

We began by investigating the downstream effects of the c-Met/β1 complex formation in breast cancer cells. To do so, we used our previously described MDA-MB-231-iDimerize-c-Met-β1 cells, which we engineered to express β1 integrin and c-Met fused to FRB (DmrC) and FKBP (DmrA), respectively, which enabled us to regulate the c-Met/β1 complex formation using AP21967 (A/C ligand heterodimerizer), a derivative of rapamycin (8). Gene expression changes induced by AP21967 treatment in MDA-MB-231-iDimerize-c-Met-β1 cells were then assessed in the NanoString nCounter platform using a 770 gene multiplex related to 13 cancer-associated canonical pathways. Genes whose expressions were upregulated by AP21967 treatment included the Wnt/hedgehog signaling genes *WNT7B* (*P* = 0.01), *ZIC2* (*P* = 0.002), and *FZD7* (*P* = 0.01; Figure 1, A and B, Supplemental Table 1, and Supplemental Figure 1; supplemental material available online with this article; https://doi.org/10.1172/jci.insight.138928DS1). Pathway analysis revealed that AP21967 treatment upregulated genes in the stem cell, TGF-β, mTOR, and Wnt signaling pathways in MDA-MB-231-iDimerize-c-Met-β1 cells (Figure 1C and Supplemental Table 2).

We then performed a more downstream assessment of the effects of c-Met/β1 complex formation in triple-negative MDA-MB-231 cells with the NanoString nCounter platform by using a different multiplex to analyze the expression of 770 genes from each step in the cancer progression process, including angiogenesis, extracellular matrix (ECM) remodeling, epithelial-mesenchymal transition (EMT), and metastasis. This analysis revealed that AP21967 treatment upregulated the expression of genes in several downstream cancer progression pathways in MDA-MB-231-iDimerize-c-Met-β1 cells, including increased HIF-1 signaling pathway and a variety of metabolic pathways (Figure 1D).

### The c-Met/β1 complex enriched the stem cell fraction in breast cancer cells.

Because of the findings of activated stem cell signaling from c-Met/β1 complex formation (Figure 1C) and because breast cancer stem cells have been shown to be a subset of breast cancer cells with enriched metastatic capacity (9), we then asked whether the ability of the c-Met/β1 complex to drive metastases (7) reflected an ability of the complex to enrich the breast cancer stem cell population. Indeed, AP21967 doubled the CD44^+^CD24^–^ stem cell fraction (10) of MDA-MB-231-iDimerize-c-Met-β1 cells (*P* < 0.001; Figure 1E and Supplemental Figure 2).

The c-Met/β1 complex drove mesenchymal gene expression in basal and luminal A breast cancer cells. AP21967 treatment also upregulated the expression of 5 of 7 assessed mesenchymal transcription factors, increasing the expression of *Twist* (*P* = 0.03) (11), *Snail* (*P* = 0.004; ref. 11), *FOXC1* (*P* = 0.02; ref. 12), *FOXC2* (*P* = 0.0003; ref. 11), and *ZEB2* (*P* = 0.04; ref. 11) in MDA-MB-231-iDimerize-c-Met-β1 cells (Figure 1F). To determine whether these effects of the c-Met/β1 complex were specific to more aggressive “basal” type triple-negative (ER, PR, and HER2 negative) breast cancer cells like MDA-MB-231, we engineered MCF7-iDimerize-c-Met-β1 cells from less aggressive MCF7 “luminal A” ER^+^PR^+^HER2^–^ breast cancer cells (Supplemental Figure 3). Inducing c-Met/β1 complex formation with A/C ligand treatment in MCF7-iDimerize-c-Met-β1 cells increased the expression of 5 of 6 assessed mesenchymal transcription factors, increasing the expression of *Twist* (*P* = 0.04), *Snail* (*P* = 0.03), *FOXC1* (*P* = 0.007), *FOXC2* (*P* = 0.002), and *ZEB1* (*P* = 0.02; Figure 1G).

### The c-Met/β1 complex promoted intravasation of triple-negative breast cancer cells.

**Figure 2:**
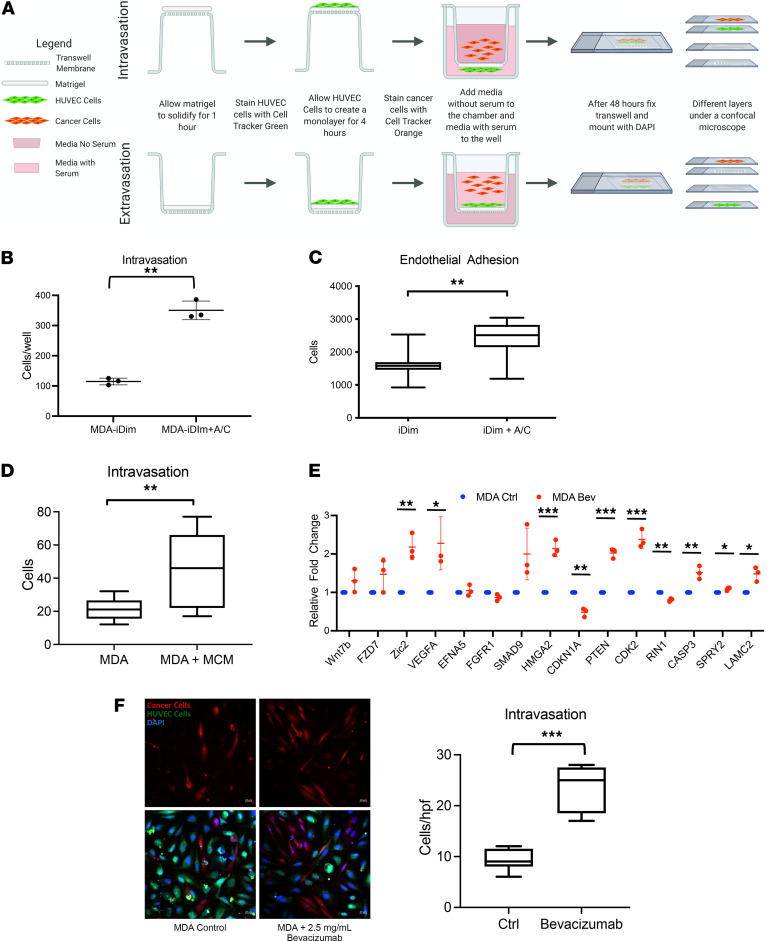
c-Met/β1 complex promotes intravasation of breast cancer cells. (**A**) Schema showing setup for cell culture assays that model breast cancer cell intravasation into circulation and extravasation out of circulation. In these assays, Matrigel and a HUVEC monolayer are plated in orientation that allows modeling of intravasation of cancer cells into circulation and extravasation of cancer cells out of circulation. Induction of c-Met/β1 complex formation in MDA-MB-231-iDimerize-c-Met-β1 cells with AP21967 treatment increased (**B**) intravasation in the cell culture intravasation assay (*n* = 3/group; scatter dot plot with horizontal line at mean and vertical line representing SD; *P* = 0.003; unpaired *t* test) and (**C**) adhesion of breast cancer cells to endothelial cells in cell culture (*n* = 9/group; whiskers = minimum/maximum; box from 25th to 75th percentile with horizontal line at median; *P* = 0.004; unpaired *t* test). (**D**) Mammosphere conditioned media (MCM) increased intravasation of MDA-MB-231 breast cancer cells (*n* = 9/group; whiskers = minimum/maximum; box from 25th to 75th percentile with horizontal line at median; *P* = 0.0098; unpaired *t* test). Bevacizumab increased (**E**) the expression of several cancer signaling pathway genes, including Wnt and hedgehog pathway gene *ZIC2* (*n* = 3/group; scatter dot plot with horizontal line at mean and vertical line representing SD; *P* = 0.003; unpaired *t* test) and (**F**) intravasation of MDA-MB-231 breast cancer cells (*n* = 8/group; whiskers = minimum/maximum; box from 25th to 75th percentile with horizontal line at median; *P* < 0.001). Scale bars: 20 μm. *P < 0.05; **P < 0.01; ***P < 0.001.

To determine the effects of the c-Met/β1 complex on intravasation of breast cancer cells, we developed a cell culture model of intravasation in which breast cancer cells were seeded in Transwell chambers and allowed to sequentially traverse Matrigel and a human umbilical vein endothelial cell (HUVEC) monolayer to model entrance into circulation (Figure 2A). This assay revealed that the c-Met/β1 complex induction with A/C ligand treatment promoted intravasation of triple-negative MDA-MB-231-iDimerize-c-Met-β1 breast cancer cells (*P* = 0.003; Figure 2B). In contrast, the induction of the c-Met/β1 complex in less aggressive “luminal” ER^+^PR^+^HER2^–^ MCF7-iDimerize-c-Met-β1 breast cancer cells did not alter the low level of intravasation exhibited by these cells (*P* = 0.9; Supplemental Figure 4). Similarly, we also found that patient brain metastases from basal triple-negative breast cancer exhibited more c-Met/β1 complex by proximity-ligation assays (PLAs) than brain metastases from luminal A, ER^+^PR^+^HER2^–^ breast cancer (*P* = 0.02; Supplemental Figure 5).

It was also noted that the induction of the c-Met/β1 complex increased the adhesion of MDA-MB-231-iDimerize-c-Met-β1 cells to endothelial cells (*P* = 0.004; Figure 2C). One potential mechanism of this c-Met/β1 complex–induced intravasation was noted when conditioned media from the breast cancer stem cells enriched by the c-Met/β1 complex induction increased intravasation of breast cancer cells in our cell culture assay (*P* = 0.0098; Figure 2D and Supplemental Figure 6). Because we previously showed that VEGF-neutralizing antibody bevacizumab treatment increased the c-Met/β1 complex formation in glioblastoma cells (7), we then analyzed the effects of bevacizumab on MDA-MB-231 cells. As with glioblastoma cells, bevacizumab increased the c-Met/β1 complex formation in MDA-MB-231 cells (Supplemental Figure 7). Bevacizumab also increased the expression of several of the pathway genes whose expressions we show to have been driven by the c-Met/β1 complex formation, including Wnt and hedgehog pathway gene *ZIC2* (*P* = 0.003; Figure 2E). In confirmation of the functional consequences of bevacizumab-induced increased c-Met/β1 complex formation and increased cancer pathway gene expression, we found that bevacizumab increased intravasation of MDA-MB-231 cells (*P* < 0.001; Figure 2F and Supplemental Figure 8).

### The c-Met/β1 complex did not promote the extravasation of breast cancer cells.

To determine the effect of the c-Met/β1 complex on the extravasation of breast cancer cells out of circulation, we modified our cell culture model of intravasation to make it a model of extravasation by seeding breast cancer cells in Transwell chambers above a HUVEC monolayer and Matrigel (Figure 2A). This assay revealed that the c-Met/β1 complex induction did not promote the extravasation of breast cancer cells (*P* = 0.8; Supplemental Figure 9).

### The c-Met/β1 complex promoted tissue-specific metastases of triple-negative breast cancer cells.

**Figure 3:**
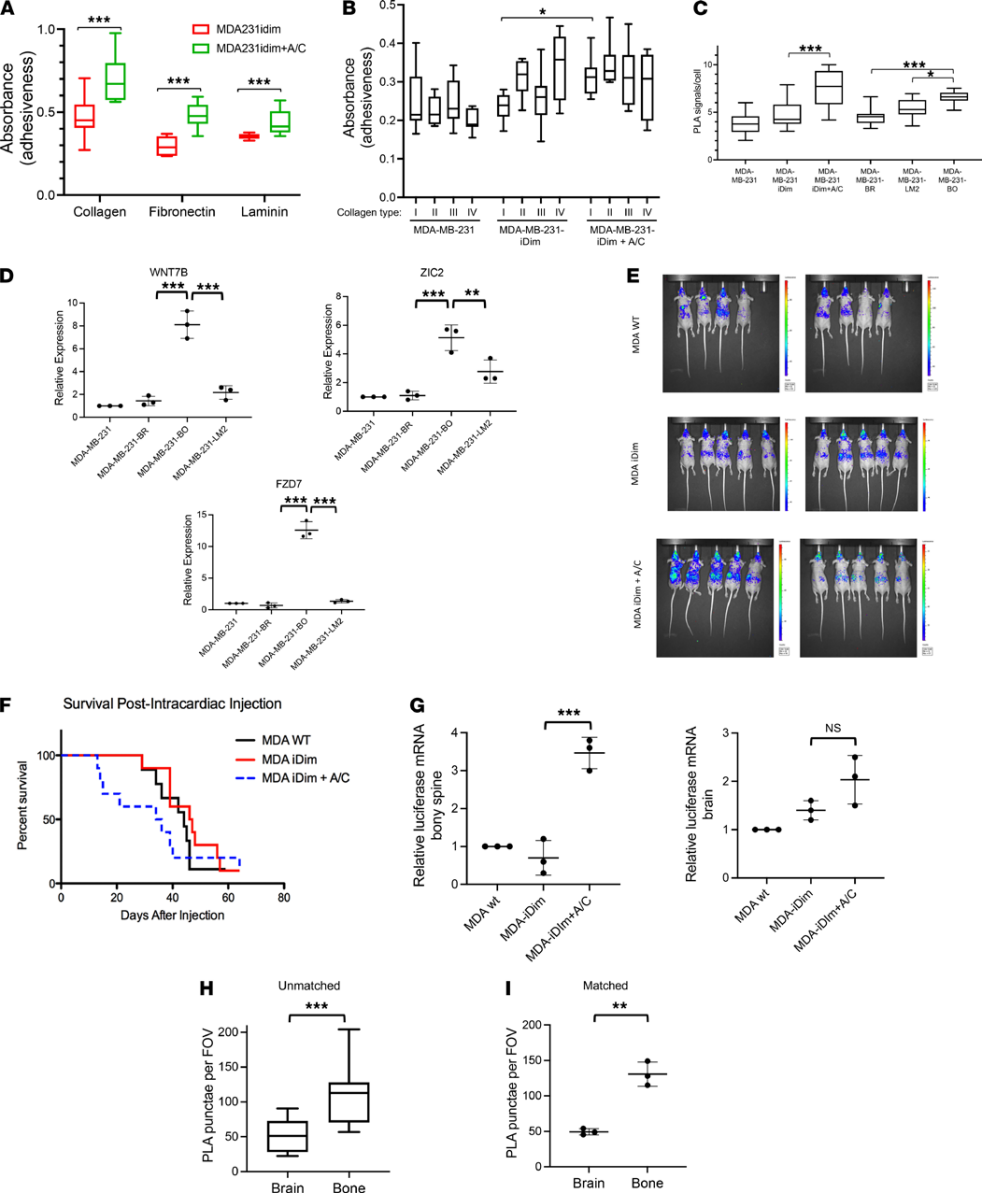
c-Met/β1 complex promotes collagen I affinity in culture and osseous metastases in vivo. Inducing c-Met/β1 complex formation in MDA-MB-231-iDimerize-c-Met-β1 promotes adhesion to (**A**) collagen, fibronectin, and laminin (*n* = 32/group; *P* < 0.001; unpaired *t* test) and (**B**) collagen I (*q* = 0.01) but not collagen II, III, or IV (*q* = 0.2–0.3; *n* = 32/group). (**C**) Proximity ligation assays (PLAs) revealed (ANOVA *P* = 0.0003) more c-Met/β1 complex in MDA-MB-231-BO cells than MDA-MB-231-LM2 (adjusted *P* = 0.03) or MDA-MB-231-BR (adjusted *P* = 0.0002) (*n* = 9/group). (**D**) Greater c-Met/β1 complex in MDA-MB-231-BO cells increased Wnt/hedgehog pathway gene expression, with increased *WNT7B* expression (ANOVA *P* < 0.0001) in MDA-MB-231-BO vs. MDA-MB-231-BR (adjusted *P* < 0.0001) or MDA-MB-231-LM2 (adjusted *P* < 0.0001); increased *ZIC2* expression (ANOVA *P* < 0.0001) in MDA-MB-231-BO vs. MDA-MB-231-BR (adjusted *P* = 0.0002) or MDA-MB-231-LM2 (adjusted *P* = 0.007); and increased *FZD7* expression (ANOVA *P* < 0.0001) in MDA-MB-231-BO vs. MDA-MB-231-BR (adjusted *P* < 0.0001) or MDA-MB-231-LM2 (adjusted *P* < 0.0001) (*n* = 3/group). (**E** and **F**) Luciferase-expressing MDA-MB-231-iDimerize-c-Met-β1 cells were pretreated in culture with AP21967 vs. vehicle, then implanted into athymic mice hearts, followed by treating mice with AP21967 or vehicle until euthanasia while monitoring for metastases by BLI. Shown are (**E**) final BLI before the first death and (**F**) Kaplan-Meier curves from the 3 groups. The complex induction via AP21967 in mice with MDA-MB-231-iDimerize-c-Met-β1 tumors shortened survival vs. vehicle (*n* = 8–10/group; *P* < 0.001; Kaplan-Meier analysis). (**G**) Organ micrometastases were detected by luciferase qRT-PCR, with more in the bony spine with AP21967 treatment of mice receiving intracardiac MDA-MB-231-iDimerize-c-Met-β1 cells compared with mice without AP21967 treatment (ANOVA *P* < 0.0001; adjusted *P* = 0.0002), but no changes in the brain (ANOVA *P* = 0.02; adjusted *P* = 0.1) (*n* = 3/group). (**H** and **I**) PLA of patient metastases revealed increased c-Met/β1 complex in osseous (*n* = 11) vs. brain metastases (*n* = 12) from (**H**) different patients (*P* < 0.001; unpaired *t* test) and (**I**) in paired bone vs. brain metastases from the same patients (*P* = 0.008; *n* = 3; paired *t* test). *P < 0.05; **P < 0.01; ***P < 0.001. Scatter dot plots: horizontal line = mean, vertical line = SD. Box-and-whisker plots: whiskers = minimum/maximum, box = 25th to 75th percentile, horizontal line = median.

We then investigated whether the c-Met/β1 complex promoted tissue-specific metastases. We began by determining if the c-Met/β1 complex promoted the adhesion to specific types of ECM proteins and found that the c-Met/β1 complex induction by AP21967 increased the adhesion of MDA-MB-231-iDimerize-c-Met-β1 cells to collagen, fibronectin, or laminin (*P* < 0.001; Figure 3A). We then assessed the effect of the c-Met/β1 complex on adhesion to purified collagen types I–IV and found that the c-Met/β1 complex induction specifically increased the adhesion of MDA-MB-231-iDimerize-c-Met-β1 cells to collagen type I (*q* = 0.01) but not collagen types II, III, or IV (*q* = 0.2-0.3; Figure 3B). We then sought to determine if the levels of the c-Met/β1 complex varied in breast cancer cells with metastatic preference for specific organs depending on the collagen content of the target organ. To do so, we used PLAs to analyze the levels of the c-Met/β1 complex in cells derived from MDA-MB-231 through serial in vivo metastases conferring affinity for specific organs (13–15). MDA-MB-231-BO bone-seeking cells had the greatest levels of complex, more than MDA-MB-231-LM2 lung-seeking (adjusted *P* = 0.03; Figure 3C) or MDA-MB-231-BR brain-seeking (adjusted *P* = 0.0002; Figure 3C) cells. These elevated levels of complex in MDA-MB-231-BO bone-seeking cells compared with MDA-MB-231-LM2 lung-seeking or MDA-MB-231-BR brain-seeking cells also led to a greater expression of the Wnt/hedgehog signaling genes we identified as associated with the complex, with increased *WNT7B* in MDA-MB-231-BO relative to MDA-MB-231-LM2 (adjusted *P* < 0.0001) or MDA-MB-231-BR (adjusted *P* < 0.0001); increased *ZIC2* in MDA-MB-231-BO relative to MDA-MB-231-LM2 (adjusted *P* = 0.007) or MDA-MB-231-BR (adjusted *P* = 0.0002); and increased *FZD7* in MDA-MB-231-BO relative to MDA-MB-231-LM2 (adjusted *P* < 0.0001) or MDA-MB-231-BR (adjusted *P* < 0.0001) (Figure 3D). These findings are consistent with the differential collagen affinities we identified because collagen type I is the predominant collagen type in bone (16) and lung (17), with greater levels in the former than the latter, whereas collagen type IV is the predominant collagen type in brain.

We then investigated the effects of the c-Met/β1 complex on tissue-specific metastases in vivo by pretreating luciferase-expressing MDA-MB-231-iDimerize-c-Met-β1 cells in culture with AP21967 or vehicle, then performing intracardiac implantation of cells, followed by serial treatment of mice with AP21967 or vehicle for the duration of mice survival while monitoring for metastases by bioluminescence (Figure 3E). We found that the c-Met/β1 complex induction via AP21967 treatment resulted in significantly shorter survival (*P* < 0.001; Figure 3F). Although there was no difference in gross metastases detected by bioluminescence of AP21967-treated mice, more micrometastases to the bony spine (*P* = 0.002), but not the brain (*P* = 0.1), were noted with AP21967 treatment based on quantitative real-time PCR (qRT-PCR) of these tissues for the luciferase gene expressed by the tumor cells (Figure 3G). Consistent with this in vivo finding and our finding that the c-Met/β1 complex induction promoted adhesion specifically to collagen type I, the levels of the complex assessed by PLAs were higher in unpaired (*P* < 0.001; Figure 3H) and paired (*P* = 0.008; Figure 3I) patient metastases to the bony spine compared with patient metastases to the brain (Supplemental Figure 10), with IP confirming this finding as well (Supplemental Figure 11).

### Genetically disrupting c-Met/β1 complex formation reduced the metastatic phenotype in triple-negative breast cancer cells.

**Figure 4:**
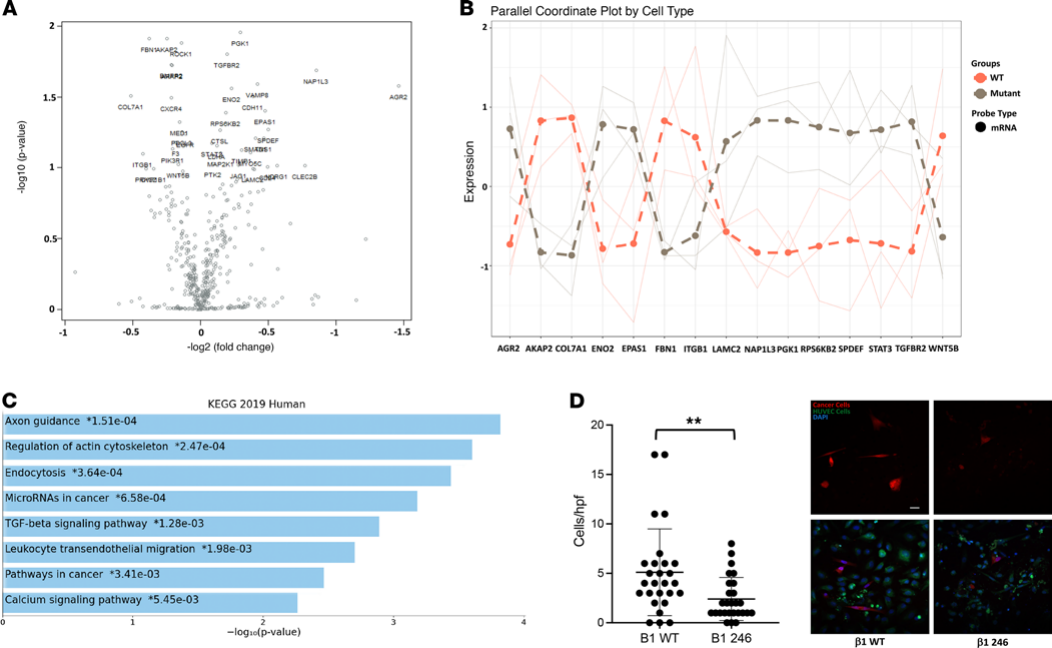
Genetic elimination of c-Met/β1 binding inhibits oncologic transcriptional changes and intravasation. Site-directed mutagenesis of β1 integrin to prevent binding to c-Met (the β1D246A mutation) in MDA-MB-231 cells altered the expression of several of the 770 cancer progression–related genes in a multiplex NanoString nCounter panel (*n* = 3/group), as evidenced by (**A**) volcano plot revealing the most upregulated genes with the largest fold change in the upper right; (**B**) top 15 hits based on *P* values; and (**C**) top upregulated pathways by *P* values based on KEGG analysis. (**D**) These gene expression changes reduced intravasation in cell culture assays of MDA-MB-231 cells with β1D246A compared with MDA-MB-231 cells with WT β1 integrin (*n* = 27/group; whiskers = minimum/maximum; box from 25th to 75th percentile with horizontal line at median; *P* = 0.003; unpaired *t* test). **P < 0.01. Scale bar: 50 μm.

Through PyMOL modeling and site-directed mutagenesis, we previously demonstrated that 5 individual amino acids in β1 integrin — 246, 283, 284, 287, and 290 — are crucial for binding of β1 integrin to c-Met (7). To determine the consequences of the lack of β1 integrin binding to c-Met, we utilized CRISPRi to knock out β1 integrin in MDA-MB-231 cells (Supplemental Figure 12) and then, via lentiviral transduction, restored WT β1 integrin or β1 integrin with change of amino acid 246 or 287 from aspartate to alanine. Either of these point mutations reduced binding of β1 integrin to c-Met (Supplemental Figure 13) and β1D246A was chosen for further investigation. Multiplex expression analysis of 770 genes from each step in the cancer progression process including angiogenesis, ECM remodeling, EMT, and metastasis revealed that, compared with cells with restored WT β1 integrin, cells with β1D246A exhibited a reduced expression of genes associated with regulating the actin cytoskeleton TGF-β signaling, and transendothelial migration (Figure 4, A–C, and Supplemental Table 3). Functional changes corresponding to these alterations in gene expression were seen when, compared with MDA-MB-231 cells with restored WT β1 integrin, cells with β1D246A exhibited reduced intravasation (*P* < 0.001; Figure 4D).

### Pharmacologically targeting the c-Met/β1 complex reduced the metastatic phenotype in triple-negative breast cancer cells.

**Figure 5:**
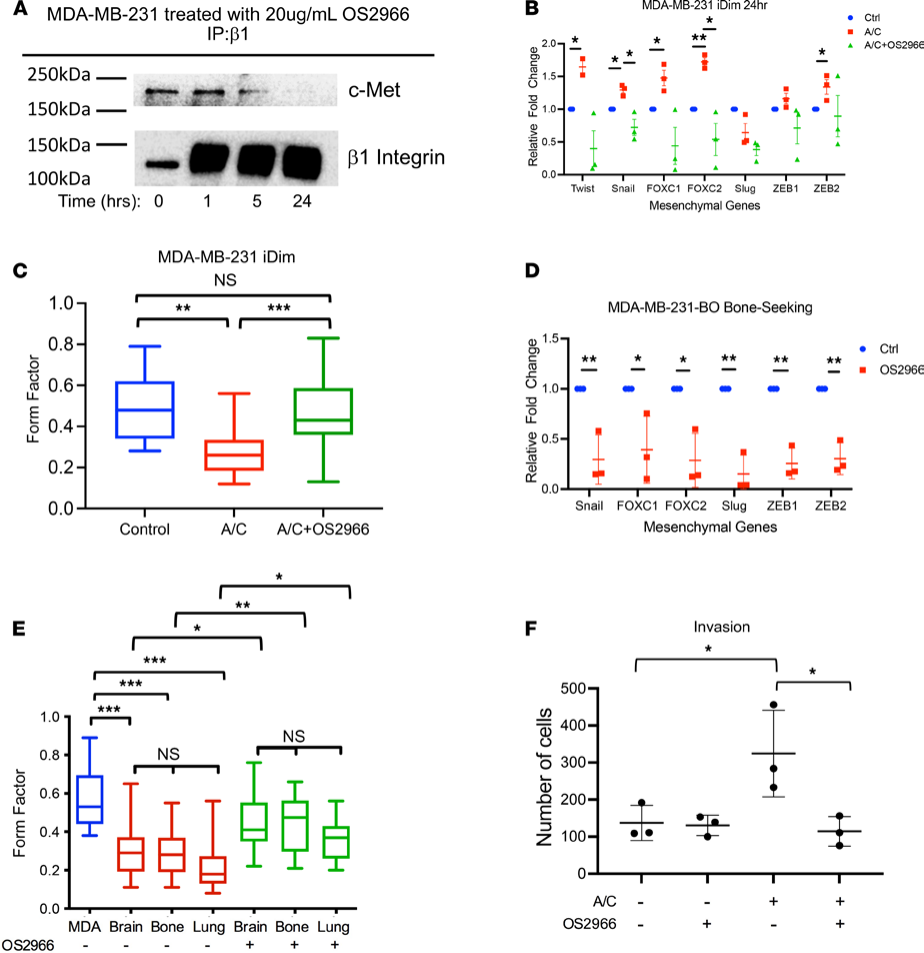
Pharmacologic disruption of c-Met/β1 binding reduced mesenchymal profile of breast cancer cells. (**A**) Pharmacologic targeting of the c-Met/β1 complex with the OS2966 antibody disrupted complex formation as evidenced by IP. (**B**) AP21967 increased the expression of 5 of 7 assessed mesenchymal transcription factors in cultured MDA-MB-231-iDimerize-c-Met-β1 cells (ANOVA *P* < 0.001; adjusted *q* values: *Twist* = 0.01; *Snail* = 0.01; *FOXC1* = 0.02; *FOXC2* = 0.002; *ZEB2* = 0.045), with 2 of these 5 changes reversed by OS2966 (adjusted *q* values: *Snail* = 0.045; *FOXC2* = 0.045) (*n* = 3/group; scatter dot plot with horizontal line at mean and vertical line representing SD). (**C**) AP21967-induced c-Met/β1 complex formation lowered the form factor of MDA-MB-231-iDimerize-c-Met-β1 cells in culture (ANOVA *P* < 0.0001; adjusted *P* = 0.001), and this effect was reversed by OS2966 (adjusted *P* = 0.0003) (*n* = 22/group; whiskers = minimum/maximum; box from 25th to 75th percentile with horizontal line at median). (**D**) OS2966 lowered mesenchymal gene expression in MDA-MB-231-BO bone-seeking cells (*Snail*: *P* = 0.008, *FOXC1*: *P* = 0.03, *FOXC2*: *P* = 0.01, *Slug*: *P* = 0.001, *ZEB1*: *P* = 0.001, and *ZEB2*: *P* = 0.002; unpaired *t* test) (*n* = 3/group; scatter dot plot with horizontal line at mean and vertical line representing SD). *Twist* expression was not detectable in these cells. (**E**) Organ-seeking cells had lower form factor than parental MDA-MB-231 cells (ANOVA *P* < 0.0001; adjusted *P* < 0.0001 for brain-, bone-, or lung-seeking vs. parental MDA-MB-231), differences that were reversed with OS2966 treatment (adjusted *P* = 0.03 for brain-seeking, 0.007 for bone-seeking, and 0.02 for lung-seeking) (*n* = 22–24/group; whiskers = minimum/maximum; box from 25th to 75th percentile with horizontal line at median). (**F**) AP21967 increased invasiveness of MDA-MB-231-iDimerize-c-Met-β1 cells in culture (ANOVA *P* = 0.02; adjusted *P* = 0.04), a change reversed by OS2966 (adjusted *P* = 0.02) (*n* = 3/group; scatter dot plot with horizontal line at mean and vertical line representing SD). *P < 0.05; **P < 0.01; ***P < 0.001.

To target the c-Met/β1 complex in breast cancer cells, we treated cells with OS2966, a therapeutic humanized β1 integrin–neutralizing antibody that we showed to inhibit the c-Met/β1 complex formation in MDA-MB-231 cells (Figure 5A). The addition of OS2966 to AP21967 offset the upregulation of 2 of the 5 mesenchymal transcription factors whose expression was increased by AP21967 in cultured MDA-MB-231-iDimerize-c-Met-β1 cells (Figure 1F): *Snail* (adjusted *q* = 0.045) and *FOXC2* (adjusted *q* = 0.045; Figure 5B and Supplemental Figure 14). Morphologic phenotypic effects associated with these transcription factor changes were also noted, as AP21967-induced c-Met/β1 complex formation lowered the form factor of MDA-MB-231-iDimerize-c-Met-β1 cells in culture (adjusted *P* = 0.001; Figure 5C), consistent with a more mesenchymal morphology, and this effect was reversed by OS2966 (adjusted *P* = 0.0003; Figure 5C).

The higher levels of c-Met/β1 complex seen in MDA-MB-231-BO bone-seeking and MDA-MB-231-LM2 lung-seeking cells (Figure 3C) were associated with higher mesenchymal gene expression in these cells (Supplemental Figure 15). Treatment with OS2966 lowered mesenchymal gene expression only in MDA-MB-231-BO bone-seeking cells (Figure 5D and Supplemental Figures 16–18). MDA-MB-231-BR, MDA-MB-231-BO, and MDA-MB-231-LM2 organ-seeking cells also had a lower form factor than parental MDA-MB-231 cells (adjusted *P* < 0.001; Figure 5E), differences that were reversed with OS2966 treatment (adjusted *P* = 0.03 for MDA-MB-231-BR, 0.007 for MDA-MB-231-BO, and 0.02 for MDA-MB-231-LM2; Figure 5E). OS2966 also reversed the invasiveness (adjusted *P* = 0.02; Figure 5F) caused by AP21967 treatment of MDA-MB-231-iDimerize-c-Met-β1 cells in culture.

## Discussion

Although cancer death rates have declined over the past decade and survival has been prolonged in several cancer types with the development of novel therapeutics, patients with metastatic disease do not share equally in these improvements (18). Despite intense efforts to understand the mechanisms underlying the metastatic cascade with the goal of uncovering effective therapeutic targets, minimal advances have been made in the treatment of metastatic cancer. For cancer patients, the majority of morbidity and mortality are associated with metastatic disease (19).

Although c-Met and β1 integrin are each known to individually contribute to metastases (20, 21), the mechanisms through which these drive metastases or invasive resistance remain uncertain because their high levels of baseline expression do not change tremendously during acquisition of metastases (20, 21). We previously addressed this knowledge gap by identifying a structural complex between c-Met and β1 integrin formed at significantly higher levels in metastatic tumors relative to their primary tumors (7). Here, we build upon that observation by determining which steps of the metastatic cascade the c-Met/β1 integrin complex drives and whether the complex promotes organ-specific metastases.

As mentioned, the metastatic cascade comprises 5 major steps: local invasion at the primary site, intravasation, extravasation, invasive colonization of the metastatic site, and proliferation at the metastatic site (3). We previously demonstrated a role for the c-Met/β1 integrin complex in invasion at the primary site but not in proliferation at the metastatic site (7). Here, we build upon that finding by showing that the c-Met/β1 integrin complex promoted intravasation, rather than extravasation, and enhanced organ-specific invasive colonization of metastatic sites, and that these processes can be not only induced by activating the c-Met/β1 integrin complex formation but also reversed by genetic or pharmacologic targeting of the complex. We also found that, although the c-Met/β1 integrin complex induced the expression of mesenchymal genes and pathways associated with metastases independent of the breast cancer’s ER/PR/Her2 receptor status, the complex formation only increased intravasation in triple-negative breast cancers, because the low baseline level of intravasation in luminal A ER^+^PR^+^HER2^–^ cells could not be enhanced by c-Met/β1 complex formation. This finding was also consistent with our observed higher levels of complex formation in brain metastases from triple-negative versus luminal breast cancer.

Our observation that the c-Met/β1 integrin complex drove intravasation of triple-negative breast cancer cells into the circulation likely reflects our demonstration of the complex promoting breast cancer cell adhesion to endothelial cells. This finding is consistent with a demonstrated role of β1 integrin in tumor cell adhesion to endothelial cells (22) via binding of tumoral α4β1 integrin to endothelial VLA-4. Because the c-Met/β1 integrin complex promotes ligand-independent conformational changes in β1 integrin that structurally resemble activation (7), it is likely that the c-Met/β1 complex promoted β1 integrin functionality in general, including VLA-4 binding. Interestingly, our demonstration that the c-Met/β1 integrin complex did not promote extravasation suggests distinct mediators of tumor cell trafficking into circulation versus out of circulation.

After extravasation, cancer cells need to find metastatic sites, which tumor cells accomplish through a variety of mediators currently being characterized (6). Our study helped address this knowledge gap because we found that triple-negative breast cancer cells with induced c-Met/β1 complex formation demonstrated organ-specific metastasis with a preference for osseous colonization. This was evident from a significantly higher burden of osseous micrometastases after in vivo c-Met/β1 complex induction, when organ-specific qRT-PCR was performed. This is likely due to preferential affinity of the c-Met/β1 complex for collagen type I, the primary ECM component of the bone. This finding of molecular alterations promoting organ-specific metastases is consistent with the observation that cancer cells target specific organs for metastases based on preferential affinity for organ-specific ECM (23). Even our understanding that cancer cells can secrete factors that can prime premetastatic niches in remote organs is based on the presumption that these tumor cell–secreted factors bring immune cells into the premetastatic niche to remodel the ECM to make it more conducive to metastases (24). This finding makes the c-Met/β1 complex an even more appealing therapeutic target because bone is the third most frequent site of metastases, with osseous metastases conferring a poor prognosis (25).

Based on our findings, the promotion of increased metastases by the c-Met/β1 integrin complex appears to be driven by the Wnt and hedgehog signaling pathways. These pathways have been implicated as mediators of metastases (26), although conflicting evidence exists as to whether they work synergistically or antagonistically in this process (27). Further work will be needed to delineate the sequence of events from the c-Met/β1 integrin complex formation that precipitates Wnt and hedgehog pathway signaling and the associated downstream events we identified such as the enrichment of stem cell fraction and mesenchymal transcription factor expression.

Our findings have significant translational implications in both preventing promotion of aggressive cancer behavior and preventing metastases. We found that bevacizumab increased the c-Met/β1 integrin complex formation in breast cancer cells, a potential mechanism of the preclinical observation that VEGF-targeted therapies (28) and bevacizumab (29) increase the metastatic potential of cancer cells. Importantly, our study demonstrates that there were viable therapeutic agents to inhibit the c-Met/β1 complex formation and its downstream events. Specifically, our demonstration that OS2966, a neutralizing antibody that disrupts the ability of β1 integrin to bind c-Met, offset the changes induced by the c-Met/β1 integrin complex formation in cultured cells is an encouraging finding that warrants further evaluation.

## Methods

### Cell culture.

MDA-MB-231 (ATCC HTB-26) and MCF7 (ATCC HTB-22) human breast adenocarcinoma cells were passaged fewer than 6 months and verified by providing sources using short-tandem repeat profiling and confirmed to be free of mycoplasma. MDA-MB-231-iDimerize-c-Met-β1 and MCF7-iDimerize-c-Met-β1 cells containing the Lenti-X iDimerize inducible heterodimer system were created as previously described (7). Complex formation was induced in these cells by treating with AP21967 (A/C ligand heterodimerizer; Takara Bio). MDA-MB-231-iDimerize-c-Met-β1/luc cells were created by lentiviral transduction of MDA-MB-231-iDimerize-c-Met-β1 cells and subsequent confirmation of bioluminescence. MDA-MB-231-BR, MDA-MB-231-BS, and MDA-MB-231-LS brain-, bone-, and lung-seeking cells were provided by Joan Massagué (Memorial Sloan Kettering Cancer Center, New York, New York, USA; refs. 13–15). Some cells expressing the iDimerize system were treated with 0.5 μM A/C ligand for the time specified in the results. Some cells were treated with 20 μg/mL of β1-neutralizing antibody OS2966 (provided by Oncosynergy) or 2.5 mg/mL of VEGF-neutralizing antibody bevacizumab (UCSF pharmacy) for 24 hours. MDA-MB-231 cells were transduced to express CRISPR/Cas9 followed by transduction with guide RNAs targeting β1 integrin, with Western blot confirming β1 knockdown. The resulting MDA-MB-231/CRISPRβ1 cells were then transduced with lentiviral vectors expressing WT β1 integrin or β1 integrin with change of amino acid 246 or 287 from aspartate to alanine (β1D246A), leading to the creation of MDA-MB-231/WT β1 and MDA-MB-231/β1D246A cells. Breast cancer cells were cultured in DMEM/F-12 supplemented with 10% FBS and 1% penicillin/streptomycin and passaged for less than 6 months. HUVECs prescreened for angiogenesis were cultured in EGM-2 (Lonza). Breast cancer cells were cultured in MammoCult (Stemcell Technologies) on non–TC-treated flasks to form mammospheres. Media collected from these cells were utilized as breast cancer stem cell–conditioned media.

### Intravasation and extravasation Transwell assays.

For intravasation, we modified a previously described protocol (30), wherein 8-μm-pore Transwell inserts (Corning) were inverted and coated with 100 μL of 6 μg/mL of growth factor–reduced Matrigel (Corning) in DPBS (Gibco) for 1 hour at room temperature. Excess Matrigel was removed, and 1 *×* 10^6^ HUVECs stained with CellTracker Green CMFDA (Invitrogen) were plated on the Matrigel-coated inverted Transwells in 100 μL of EGM-2 for 4 hours in a 37°C CO_2_ incubator to create a monolayer as previously described (30). Transwells were then flipped right side up into a 24-well plate (Corning), and 30,000 tumor cells stained with CellTracker Orange CMRA (1:1000; Invitrogen) were added. Transwells were washed with PBS and fixed in 4% paraformaldehyde (PFA)/PBS for 15 minutes. Inserts were mounted on a slide with DAPI Fluoromount-G (Southern Biotech). Only tumor cells that breached the endothelial monolayer were scored as a positive transendothelial migration event. Nine fields of view were acquired for each Transwell (original magnification, ×20).

For extravasation, we modified a previously described protocol (31), wherein 8-μm-pore FluoroBlok Transwell inserts (Corning) were coated inside the chamber with 100 μL of 6 μg/mL of growth factor–reduced Matrigel in DPBS for 1 hour at room temperature. Excess Matrigel was removed, and 1 *×* 10^6^ HUVECs stained with CellTracker Green CMFDA were plated on the Matrigel-coated FluoroBlok Transwells in 100 μL of EGM-2 for 4 hours in a 37°C CO_2_ incubator, and 30,000 tumor cells stained with CellTracker Orange CMRA were added. Transwells were washed with PBS and fixed in 4% PFA/PBS for 15 minutes. Inserts were mounted on a slide with DAPI Fluoromount-G. Only tumor cells that were on a different plane from the endothelial monolayer were scored as a positive extravasation event. Nine fields of view were acquired for each Transwell (original magnification, ×20; ref. 31).

### HUVEC adhesion assays.

Forty-eight–well plates were coated with Matrigel as described above. HUVECs were seeded at a density of 50,000 cells per well and grown to confluence. Tumor cells were stained with CellTracker Green CMFDA. EGM-2 media were removed and DMEM/F-12 was added for the remainder of the assay, and 25,000 tumor cells were added to each well and incubated to allow the adhesion to the HUVEC monolayer. After 30 minutes, media were removed and wells were washed 3 times with DPBS to remove any unbound tumor cells. Wells were fixed in 4% PFA/PBS for 15 minutes. Three fields of view were acquired for each well (32).

### qRT-PCR.

RNA extraction was performed using either Quick-RNA Miniprep Kit (Zymo Research) or RNeasy Mini Kit (QIAGEN). Complementary DNA (cDNA) was synthesized using the qScript cDNA XLT SuperMix (Quantabio). qRT-PCR using SYBR Green (Quantabio) was performed via QuantStudio 3 System (Applied Biosystems). Primers are listed in Supplemental Table 4.

### PLAs.

The Duolink In Situ Red Starter Kit Mouse/Rabbit (Sigma-Aldrich) was used to assess the c-Met/β1 complex formation using antibodies summarized in Supplemental Table 5 as previously described (7).

### Western blotting.

Human tissue samples and cell preparations were harvested in complete 1× radio immunoprecipitation buffer made from a 10× stock solution (9806, Cell Signaling Technology) and 1 tablet each of PhoStop and Complete Mini (04906845001 and 04693124001, Roche). Insoluble materials were removed by centrifugation at 21,000*g* for 15 minutes at 4°C. Protein concentration was determined using the bicinchoninic acid assay (23225, Thermo Fisher Scientific). Samples were prepared with 10–30 μg of protein in RIPA buffer with 4× LDS loading buffer (LP0001, Life Technologies). Samples were electrophoresed on SDS-PAGE gels, transferred to PVDF membranes, and probed with primary antibodies (Supplemental Table 5) overnight at 4°C. Membranes were detected using HRP-conjugated secondary antibodies and imaged using radiographic film or the Odyssey Fc Imaging System (LI-COR).

### Immunoprecipitation.

Samples were prepared in 500 μL of complete RIPA buffer containing 1000–1500 μg of protein. For β_1_ IP, 50 μL of Protein A Magnetic Beads slurry (73778S, Cell Signaling Technology) were aliquoted per sample and washed with 500 μL of complete RIPA buffer. Beads were incubated with rabbit monoclonal anti-β_1_ antibody (1:40; ab52971, Abcam) in 400 μL of 0.1% Triton X-100 PBS for 15 minutes at room temperature. Beads were magnetically precipitated and resuspended with sample lysate for incubation on a rotator (4°C overnight). Antibody-bound beads were magnetically separated from the lysate supernatant and washed 3 times in 500 μL of Pierce IP Lysis Buffer (Thermo Fisher Scientific). Samples were eluted by precipitating beads on a magnetic rack and resuspending in 40 μL of 3× Blue Sample Buffer 30× Reducing Agent: 1.25 M DTT (1:30; 7722s, Cell Signaling Technology). Resulting samples were heated at 95°C (5 minutes), centrifuged at 300*g* at room temperature (1 minutes), and magnetically precipitated. The supernatant (20 μL) was used for each SDS-PAGE electrophoresis. Blots were probed with primary and secondary antibodies (Supplemental Table 5).

### Flow cytometry.

MDA-MB-231-iDimerize-c-Met-β1 cells were treated with either 0.5 mM A/C ligand or the equivalent dilution (1:1000) of 100% ethanol for 3 hours. The cells were then washed with PBS and dissociated with TrypLE (Thermo Fisher Scientific) and washed with PBS. Cells (1 *×* 10^6^) were resuspended in 100 μL of PBS plus 10% FBS and stained with anti-CD44–APC (Biolegend) and anti-CD24–FITC (Biolegend) or isotype controls at 4°C for 30 minutes. The samples were washed with PBS once and resuspended in 1 mL PBS plus 10% FBS. Flow cytometry was performed via Sony SH800 and data were analyzed using FlowJo.

### Animal work.

Athymic mice (The Jackson Laboratory) were anesthetized using isoflurane and buprenorphine injected i.p. at a dose of 0.1 mg/kg. The injection area was prepared with Betadine solution. Intracardiac inoculation was performed by passing a 30-gauge × 0.5-inch needle into the left ventricle. Proper location was confirmed by presence of arterial blood pulsating into the needle, and 1 × 10^5^ MDA-MB-231-iDimerize-c-Met-β1/luc cells suspended in 100 μL serum-free media were injected into the left ventricle. Serial whole-body bioluminescence imaging (BLI) was performed to track systemic metastases.

### Cell morphology (form factor).

Cells were plated on 8-well Lab-Tek II Chamber Slides. Twenty-four hours later, media were aspirated, and the slides were fixed in 4% PFA for 15 minutes. The slides were then washed 3 times in PBS for 5 minutes. The slides were then incubated in 6.6 μM Phalloidin (Cell Signaling Technology) diluted 1:20 in PBS for 15 minutes at room temperature. Subsequently,m the slide was washed in PBS once and mounted with DAPI Fluoromount-G. Slides were imaged (original magnification, ×20) and form factor was analyzed using the Shape Descriptors plugin on NIH ImageJ software.

### Nanostring multiplex transcriptomic analysis.

Using the RNeasy Mini kit (QIAGEN), RNA was extracted from (a) MDA-MB-231-iDimerize-c-Met-β1 cells treated with and without AP21967 for 3 hours and (b) MDA-MB-231/CRISPRβ1 cells transduced to express WT β1 integrin or β1 integrin with change of amino acid 246 to alanine. A bioanalyzer was used to determine the quantity and quality of the RNA sample. RNA (100 ng) was used for the Cancer Pathways Panel and 175 ng of RNA was used for the Cancer Progression Panel. RNA from each sample was hybridized with the codeset for 18 hours, and 30 μL of the reaction was loaded into the nCounter cartridge and run on the nCounter SPRINT Profiler. Enrichr software (https://maayanlab.cloud/Enrichr/) was used to analyze the expression of pathways defined in the KEGG 2019 Human database and their statistical significance using the differentially expressed genes were obtained from the Nanostring nSolver software analysis as input.

### Statistics.

For comparing continuous variables, 2-tailed *t* test (parametric) or Kruskal-Wallis/Wilcoxon rank sum test (nonparametric) was used, with analysis using SPSS (IBM, v24.0). Multiple comparisons involving multiple groups compared for a single variable were analyzed by 1-way ANOVA and Tukey’s test for multiple testing of nonparametric data (GraphPad Prism 9). For multiple comparisons involving multiple groups compared for multiple variables, FDR-adjusted *q* values were calculated. Kaplan-Meier analysis was used to compare survival of mice treatment groups. Experiments were done with 3 technical and 3 biologic replicates. Error bars are SDs among biologic replicates. The threshold for statistical significance was *P* < 0.05 or FDR-adjusted *q* < 0.05.

### Study approval.

Human tissue research was approved by the IRB of UCSF (approval number 11-06160). Animal experiments were approved by the IACUC of UCSF (approval number AN105170-02).

## Author contributions

DL, HW, SS, and ACCC designed the project, conducted the experiments, processed the data, and interpreted the results. SJ conducted the experiments, processed the data, interpreted the results, and edited the figures. AC, ATN, and AJ developed plasmids, cell lines, and experimental techniques. JMS processed the data and interpreted the results. AP conducted the experiments and processed the data. SSS, JC, MMS, and GY conducted the experiments, processed the data, and interpreted the results. MKA was responsible for overall conception of the project, including procuring funding, developing experimental design, interpreting the results, and writing and editing the manuscript.

## Supplementary Material

Supplemental data

## Version 1 05/18/2021

In-Press Preview

## Version 2 06/22/2021

Electronic publication
