# Supplementary material for: Role of c-Met/β1 integrin complex in the metastatic cascade in breast cancer
Source: JCI Insight. 2021 Jun 22;6(12):e138928. doi: 10.1172/jci.insight.138928 (PMC8262466; doi:10.1172/jci.insight.138928)
Supplement: Supplemental data [file jciinsight-6-138928-s006.pdf]

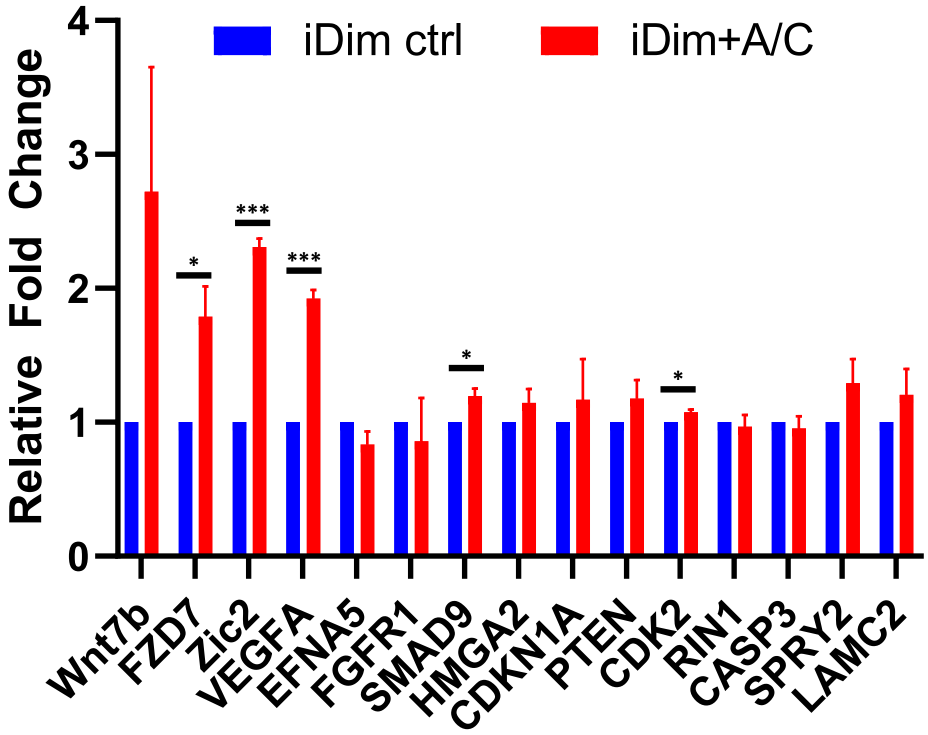

Supplemental Figure 1. Validation of multiplex transcriptomic results by qPCR confirms elevated Wnt/hedgehog pathway signaling after c-Met/β1 complex formation. Related to Figures 1A-B. qPCR was performed to validate results obtained from multiplex transcriptomic analysis. A/C ligand increased expression of Fzd7 (P=0.03), Zic2 (P<0.001), VEGFA (P<0.001), Smad9 (P=0.03), and CDK2 (P=0.03). n=3/group. *P<0.05, **P<0.01, ***P<0.001.

Supplemental Figure 2. Confirming that mammospheres express breast cancer stem cell genes. Related to Figure 1E. Use of qPCR to verify that mammospheres derived from MDA-MB-231 breast cancer cells expressed breast cancer stem cell genes at higher levels than adherent MDA-MB-231 cells. Shown are individual results from the five stem cell genes. n=3/group. *P<0.05; **P<0.01; ***P<0.001.

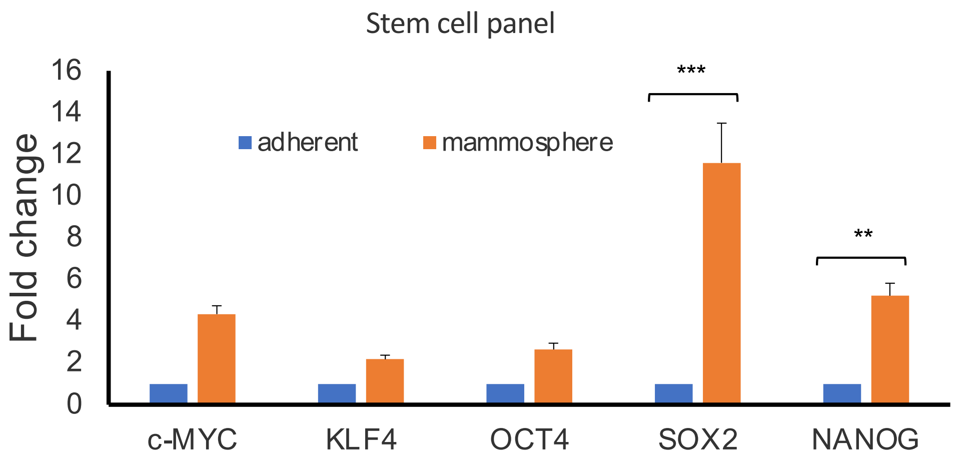

Supplemental Figure 3. Assessment of c-Met/β1 complex formation in MCF7-iDimerize-c-Met-β1 cells. Related to Figure 1G. (A) Co-Immunoprecipitation showing gradual increase in complex formation upon addition of Ac ligand. Exogenous ITGB1 was pulled down using HA. (**B**) Endogenous Immunoprecipitation of ITGB1 shows gradual increase in complex formation.

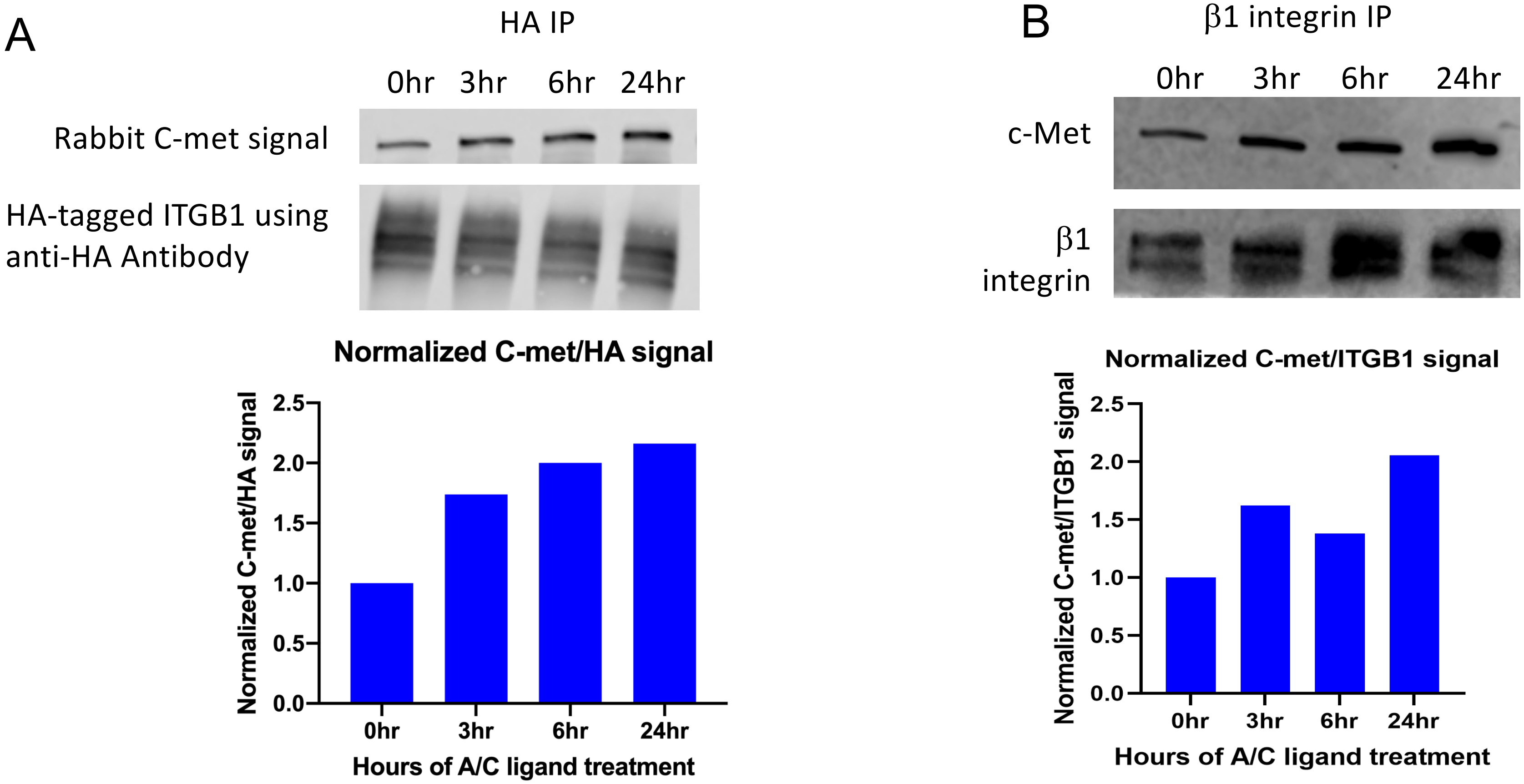

Supplemental Figure 4. Induction of c-Met/β1 complex formation in MCF-7 cells does not promote intravasation of breast cancer cells. Related to Figure 2B. Shown are immunostainings of CMRA-labeled luminal A MCF-7 breast cancer cells which were incubated in a cell culture intravasation assay for 48 hours in the absence or presence of A/C ligand. n=3/group. Scale bar, 50 μm.

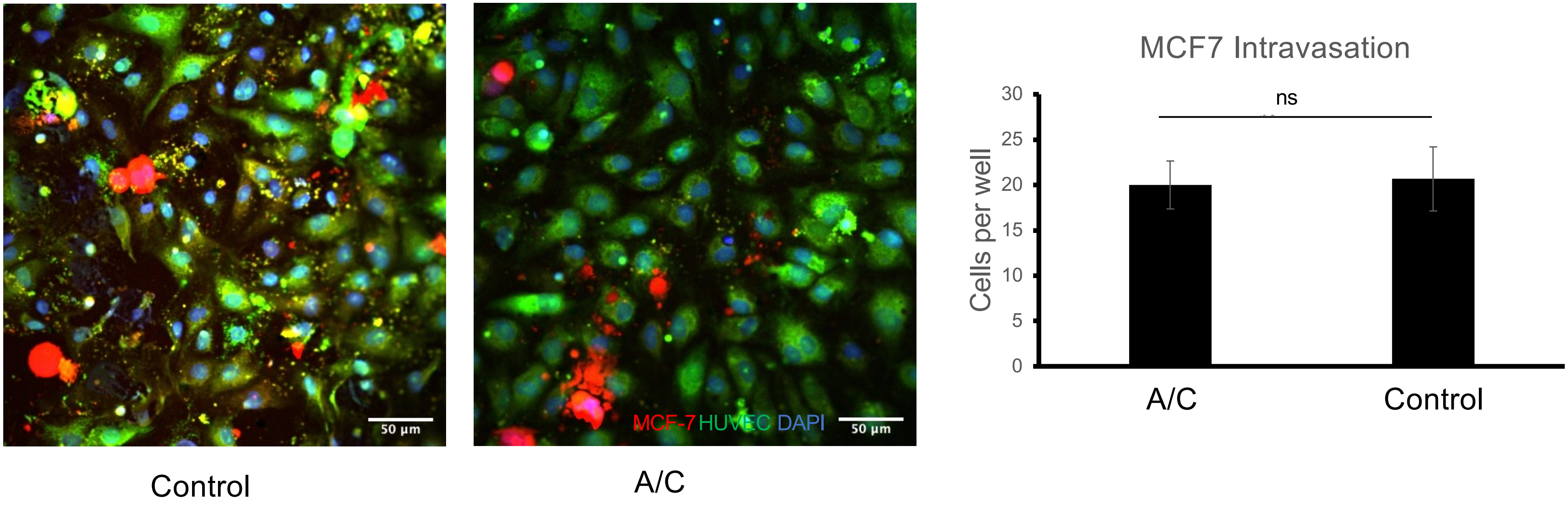

Supplemental Figure 5. Increased c-Met/β1 complexes detected in brain metastases derived form triple-negative versus luminal breast cancer. Related to Figure 2B. Shown are example PLA results for patient breast cancer brain metastases, revealing more c-Met/β1 complex in brain metastases from triple-negative versus luminal breast cancer (P=0.02; n=7).

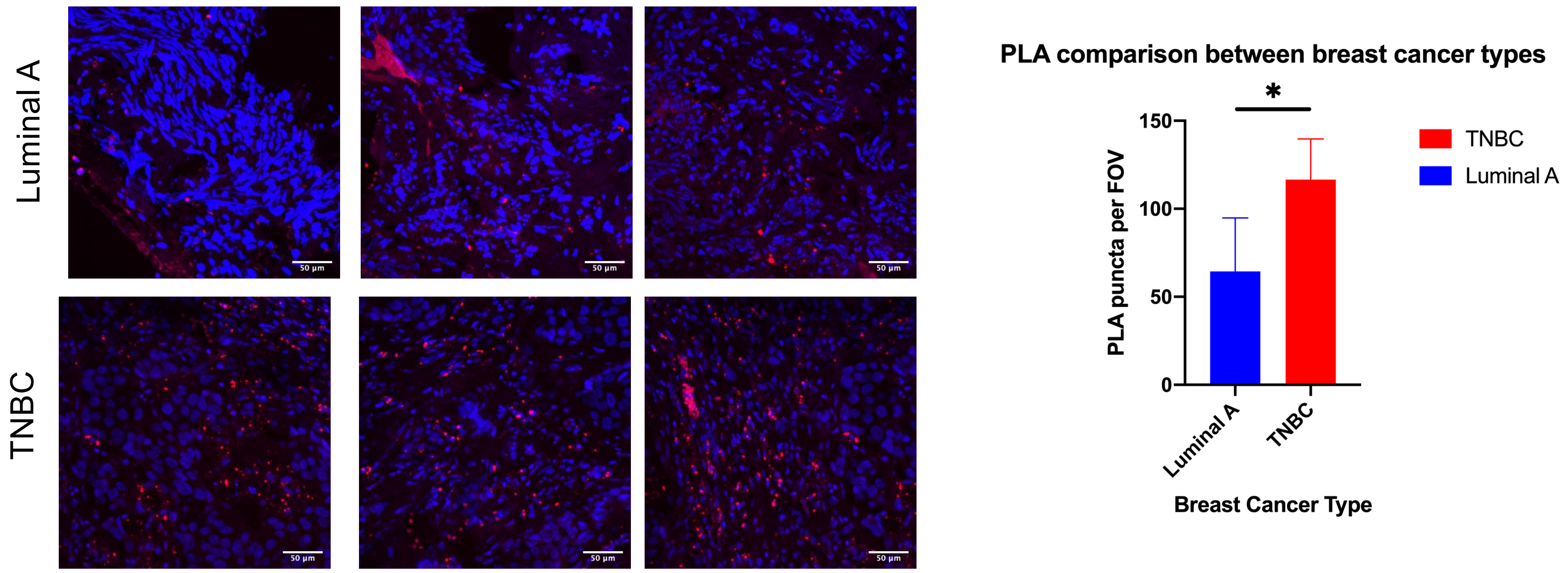

Supplemental Figure 6. Mammosphere conditioned media promotes intravasation of breast cancer cells. Related to Figure 2D. Shown are immunostainings of CMRA-labeled MDA-MB-231 breast cancer cells which were incubated in a cell culture intravasation assay for 48 hours in the absence or presence of mammosphere-conditioned media (MCM). Results are quantified in **Figure 2D**. n=3/group. Scale bar, 200 μm.

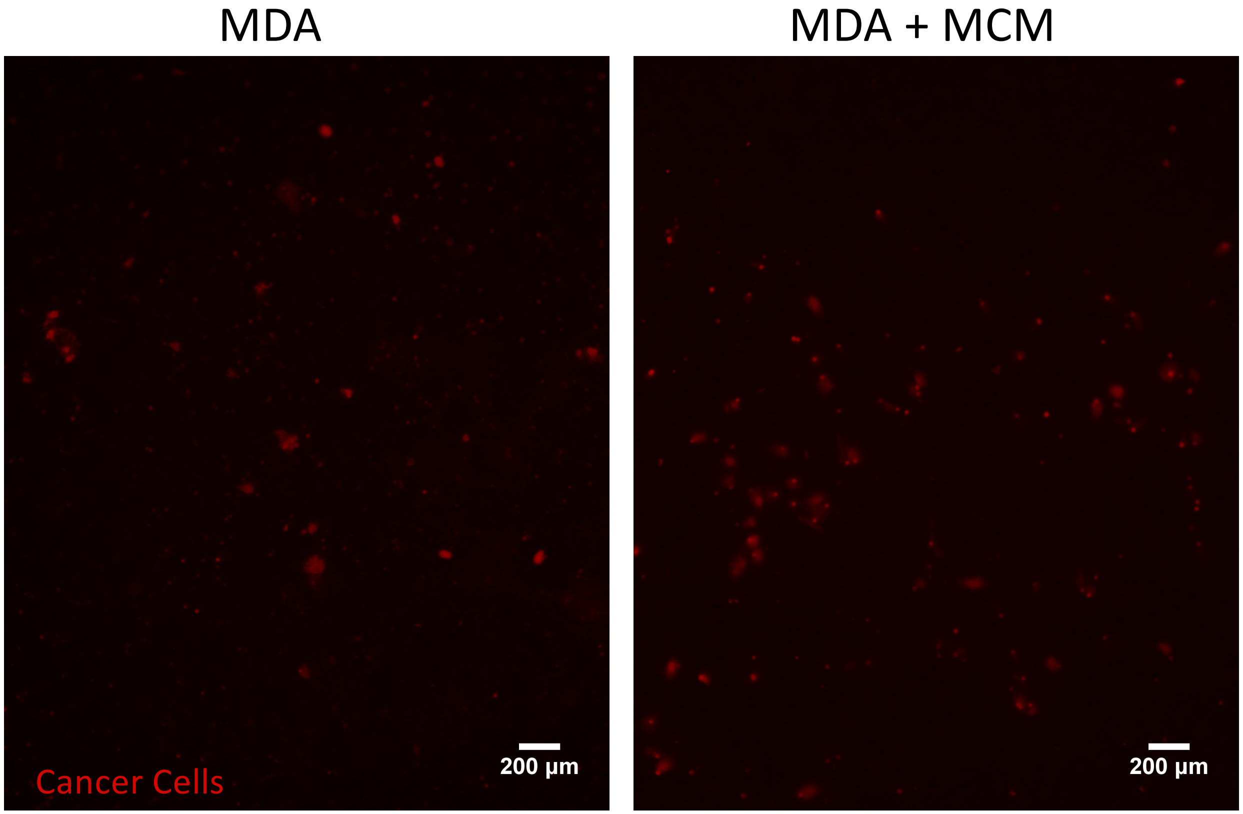

Supplemental Figure 7. Bevacizumab increases c-Met/β1 complex formation in breast cancer cells. Related to Figs. 2E-F. Immunoprecipitation to pull down β1 integrin revealed more c-Met/β1 complex formation after treating MDA-MB-231 breast cancer cells with 2.5 mg/mL bevacizumab for 24 hours.

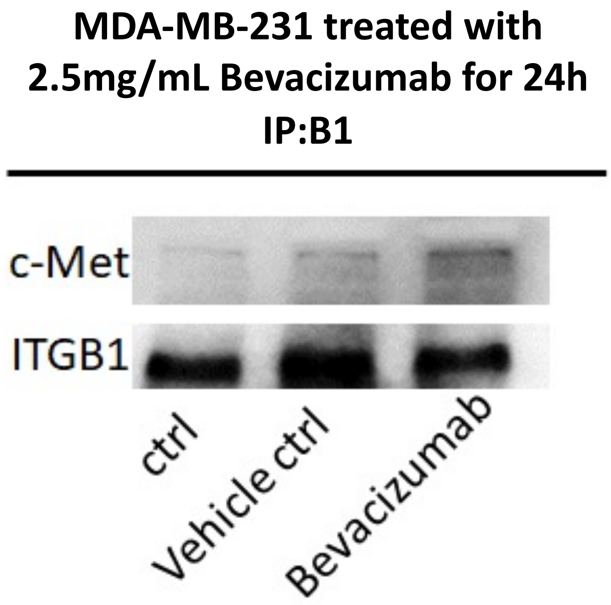

Supplemental Figure 8. Bevacizumab increases intravasation of breast cancer cells. Related to Figure 2F. Shown are immunostainings of CMRA-labeled MDA-MB-231 breast cancer cells and CMFDA-labeled HUVEC cells with DAPI nuclear staining in blue at the 48 hour time point after intravasation assays. n=3/group. Results are quantified in **Figure 2F**. Scale bar, 20 μm.

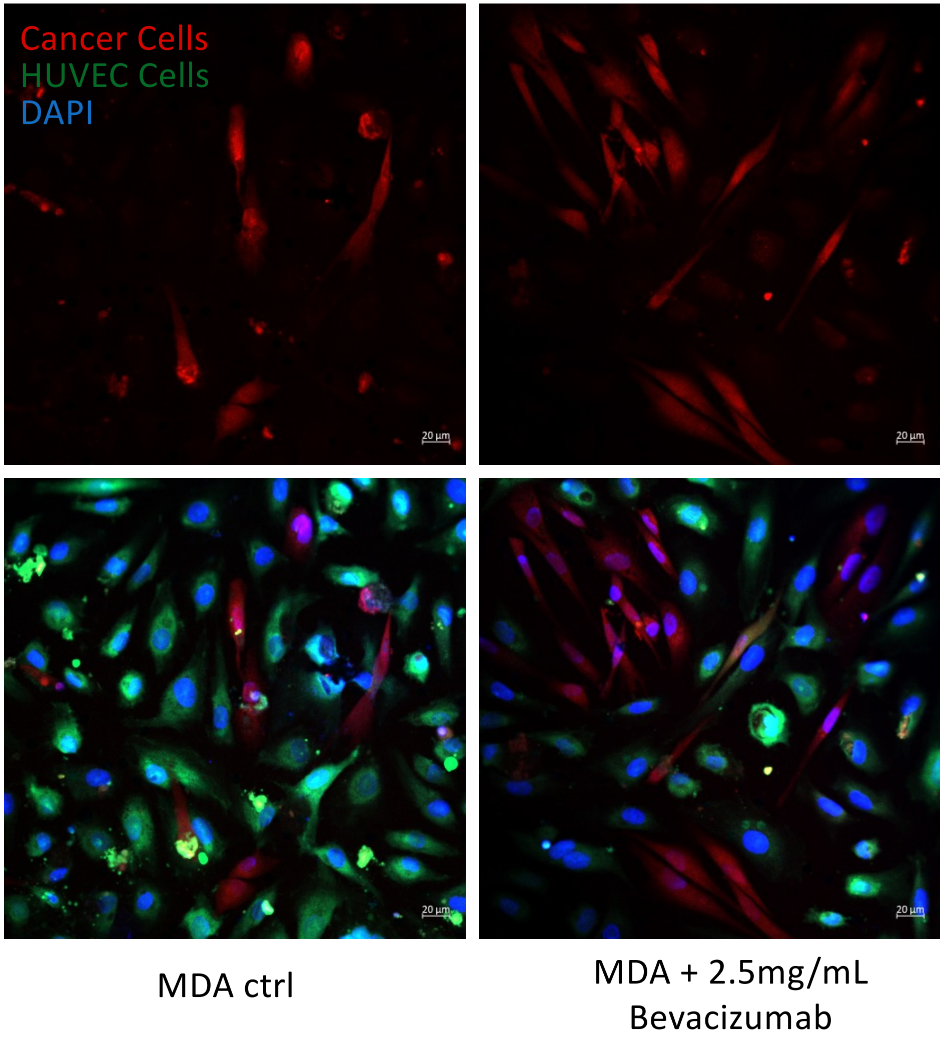

Supplemental Figure 9. c-Met/β1 complex does not alter extravasation of breast cancer cells. Related to Fig. 2. Induction of Shown are immunostainings of CMRA-labeled MDA-MB-231 cells at the 48 hour time point after a cell culture extravasation assay.

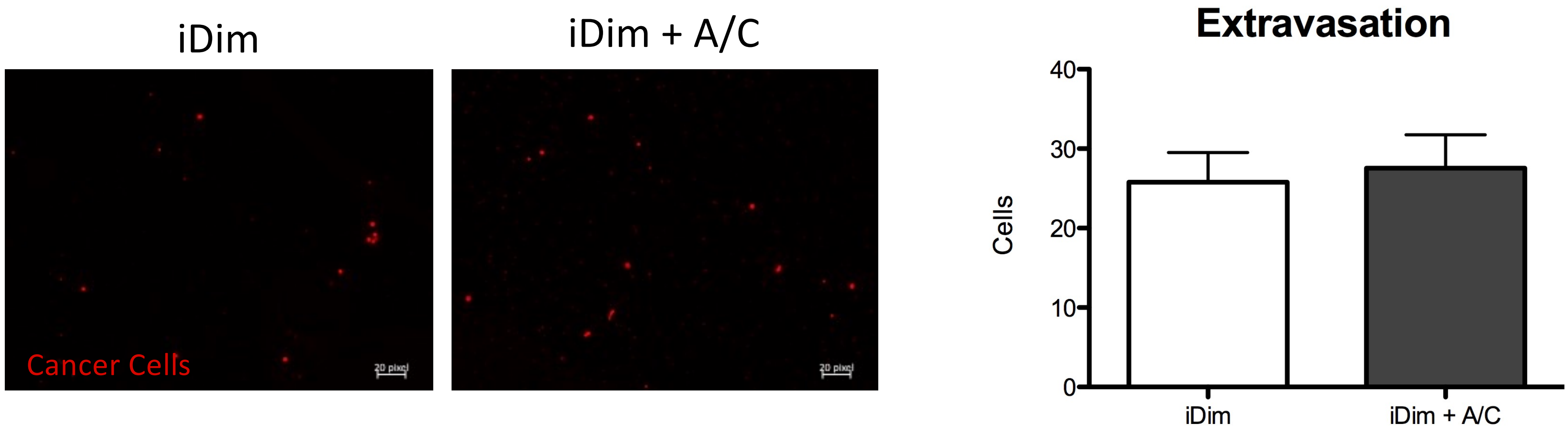

Supplemental Figure 10. Proximity-ligation assays of patient samples reveal c-Met/β1 complexes in different types of metastases. Related to **Figure 3G.** Shown are PLA immunostainings of breast, renal cell cancer (RCC), and prostate cancer metastases to brain versus bony structures. Results were quantified in **Figure 3G**.

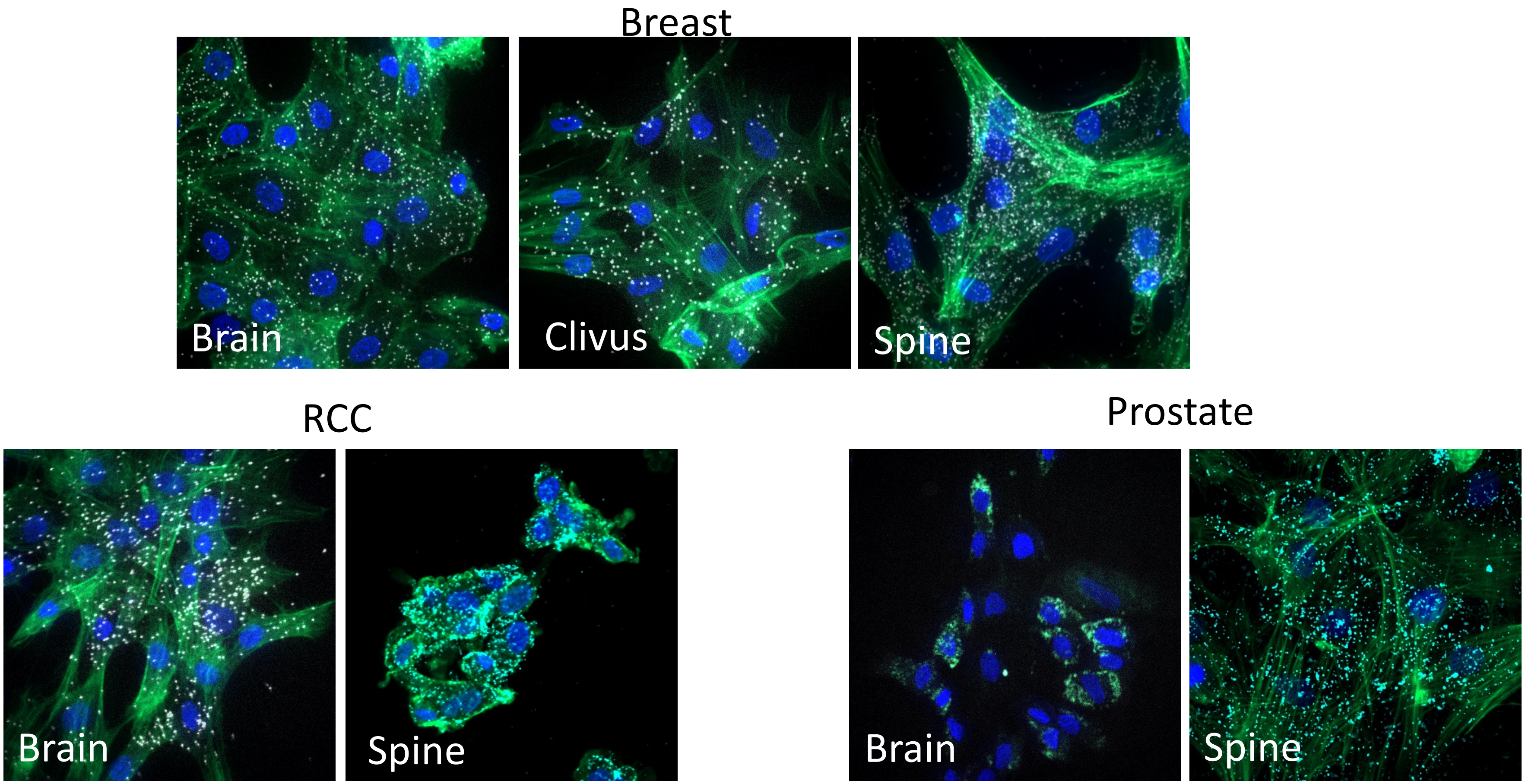

Supplemental Figure 11. Immunoprecipitation of patient samples reveals c-Met/β1 complexes in different types of metastases. Related to Fig. 3G. Shown are immunoprecipitations of human tumors in which c-Met is precipitated and blotted for β1 integrin. The ratio of β1 integrin to c-Met quantified band intensity was higher in patient bony metastases (n=11) relative to brain metastases (n=12) from different patients (left two bars; $P<0.01$) and in paired bone metastases relative to brain metastases from the same patients (n=3; right panel; $P<0.001$). *$P<0.05$; **$P<0.01$; ***$P<0.001$.

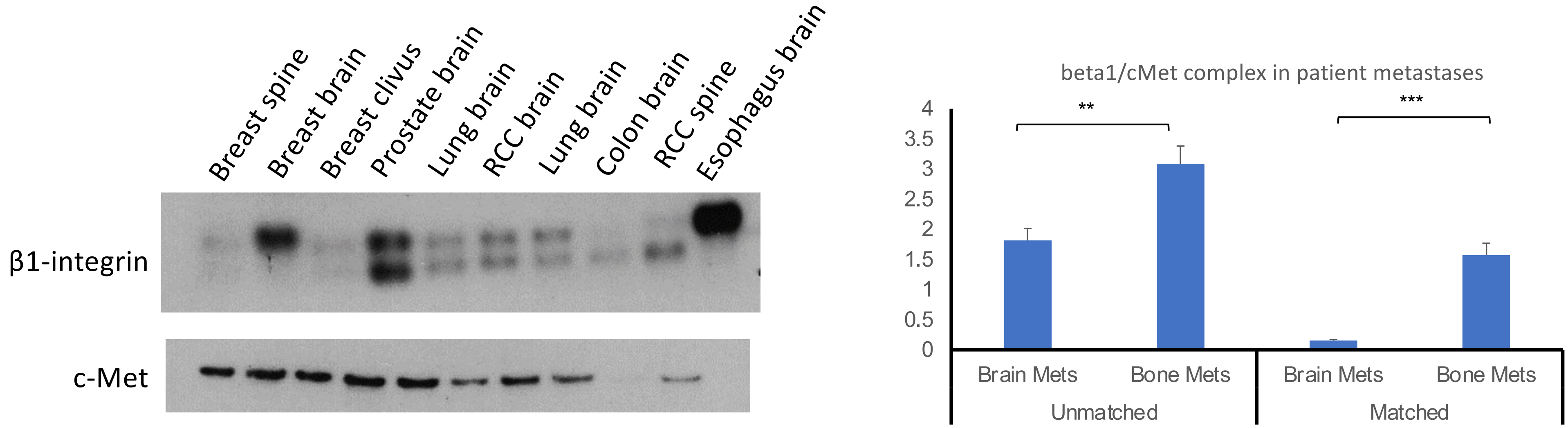

Supplemental Figure 12. β1 integrin knockdown via CRISPRi in breast cancer cells. Related to Fig. 4. MDA-MB-231 breast cancer cells were engineered to express KRAB CAS, followed by guide RNAs targeting β1 integrin. Western blot revealed loss of β1 integrin expression in the resulting cells.

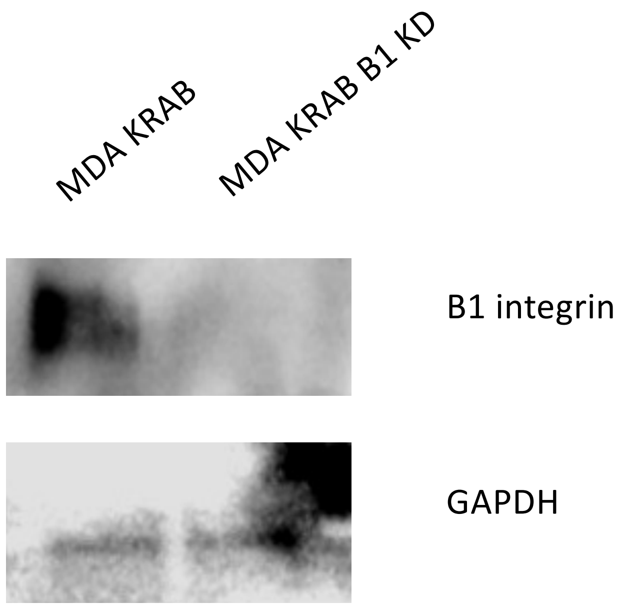

Supplemental Figure 13. Immunoprecipitation reveals point mutations that lower binding of β1 integrin to c-Met. Related to Fig. 4. Shown are results when immunoprecipitating for β1 integrin and blotting for c-Met as well as confirmatory blot for β1 integrin in MDA-MB-231 cells engineered for β1 integrin loss via CRISPR followed by restoration of wild-type β1 integrin (ctrl) or restoring β1 integrin with point mutations D246A and D287A which we previously demonstrated to reduce binding to c-Met in glioblastoma cells. Results confirmed reduced binding to c-Met in MDA-MB-231 breast cancer cells.

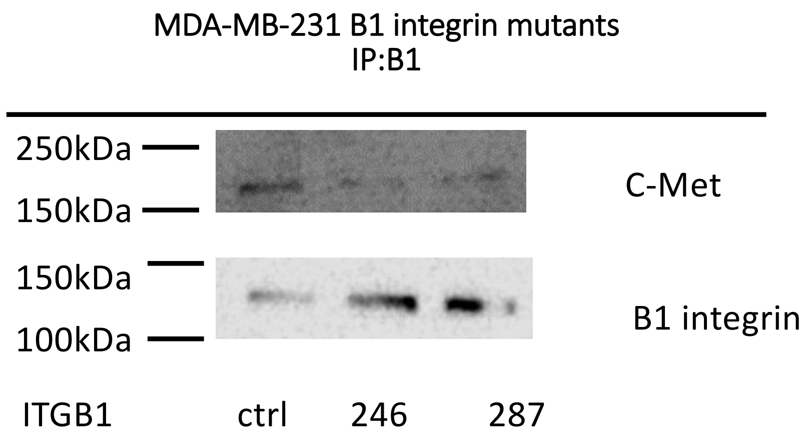

Supplemental Figure 14. Individual mesenchymal transcription factors do not change in expression after OS2966 treatment of MDA-MB-231 iDim cells. Related to Figure 5B. Shown are qPCR results for six mesenchymal transcription factors in MDA-MB-231-iDimerize-c-Met-β1 cells after 24 hours of OS2966 treatment (n=3/group). *P<0.05; **P<0.01; ***P<0.001.

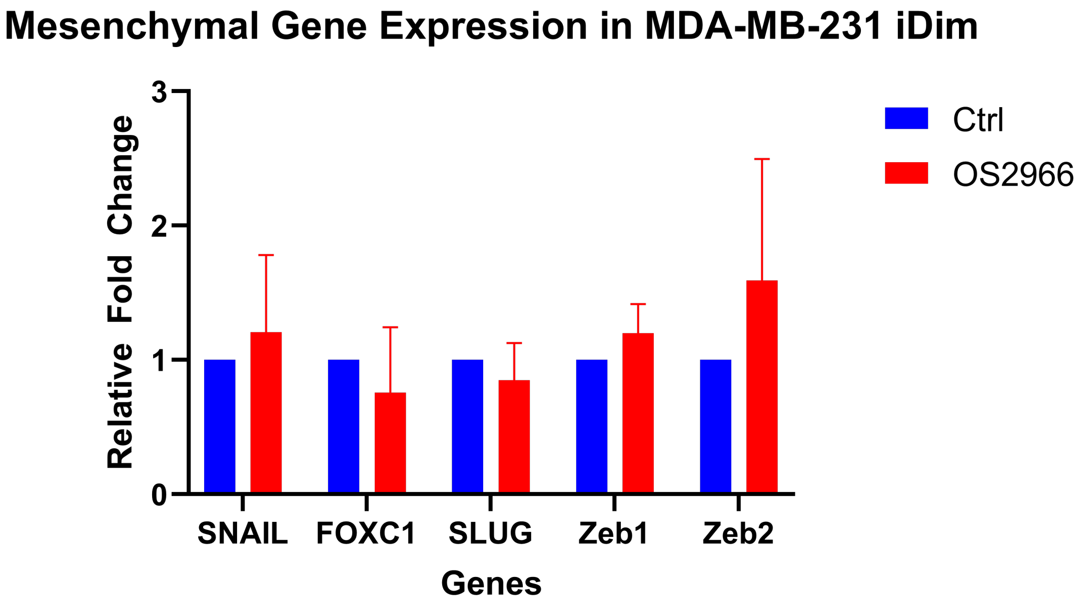

Supplemental Figure 15. Bone and lung-seeking breast cancer cells have elevated expression of mesenchymal genes compared to brain-seeking and parental breast cancer cells. Related to Figure 5D. Shown are relative expression of seven mesenchymal genes in MDA-MB-231 parental breast cancer cells and MDA-MB-231-BR brain-seeking, MDA-MB-231-BO bone-seeking, and MDA-MB-231-LM2 lung-seeking cells derived from the parental MDA-MB-231 cells. *P<0.05; **P<0.01; ***P<0.001.

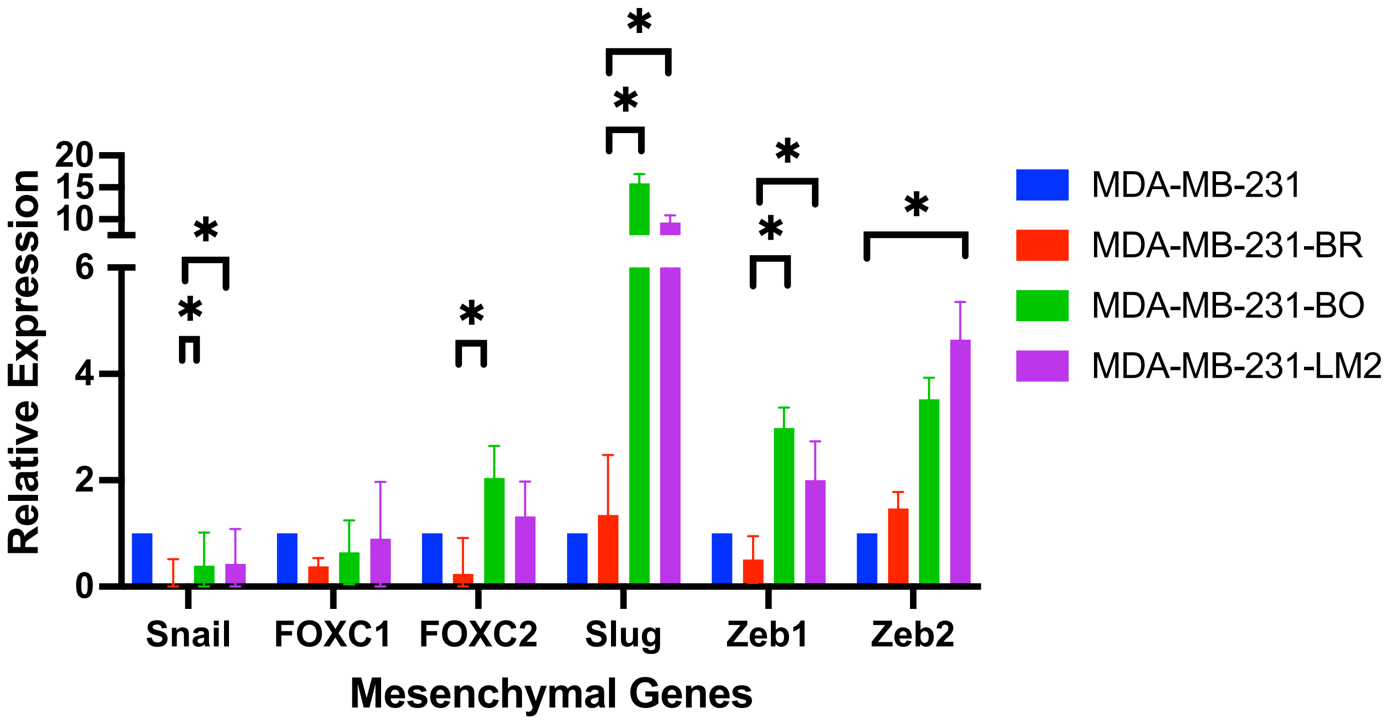

Supplemental Figure 16. Individual mesenchymal transcription factors do not change in expression after OS2966 treatment of MDA-MB-231 breast cancer cells. Related to Figure 5D. Shown are qPCR results for six mesenchymal transcription factors in MDA-MB-231 lines after 48 hours of OS2966 treatment (n=3/group). *P<0.05; **P<0.01; ***P<0.001.

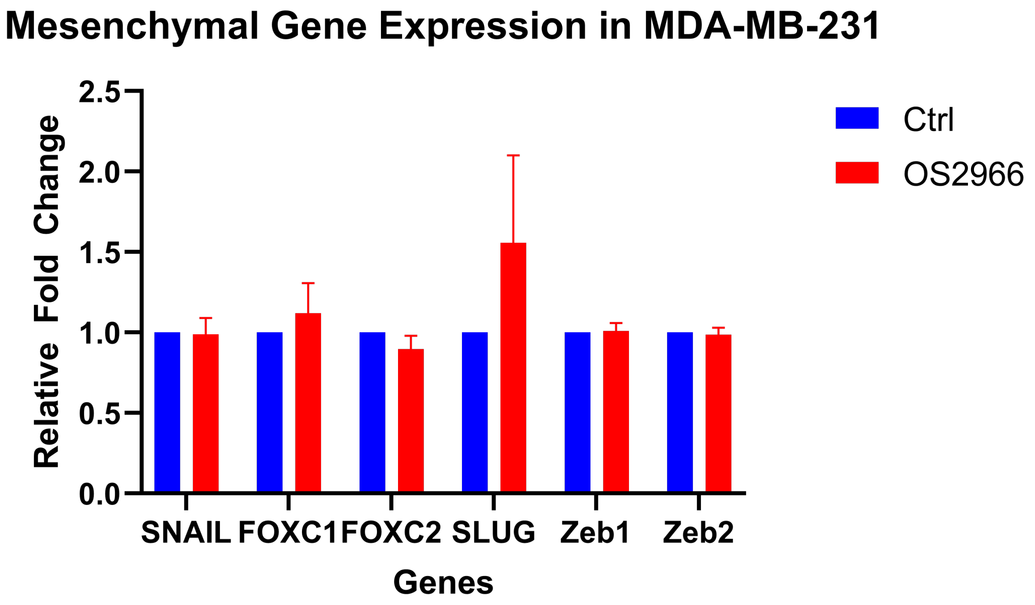

Supplemental Figure 17. Individual mesenchymal transcription factors do not change in expression after OS2966 treatment of MDA-MB-231-BR brain-seeking breast cancer cells. Related to Figure 5D. Shown are qPCR results for six mesenchymal transcription factors in MDA-MB-231 BR lines after 48 hours of OS2966 treatment (n=3/group). *P<0.05; **P<0.01; ***P<0.001.

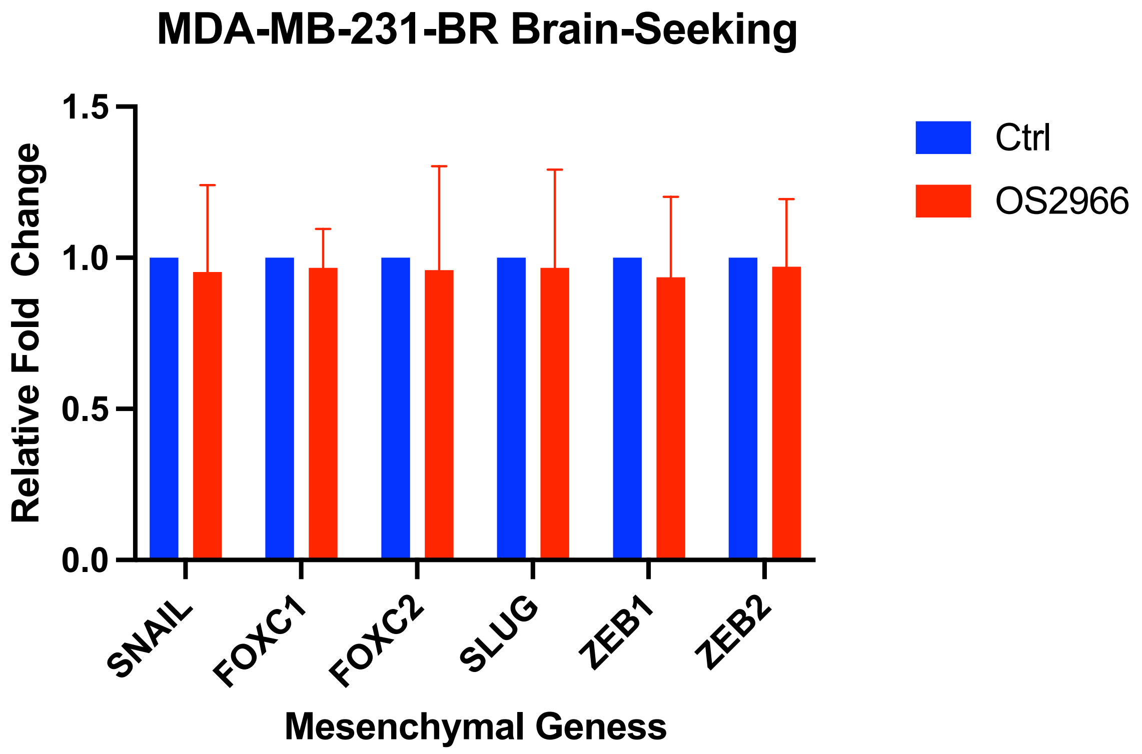

Supplemental Figure 18. Individual mesenchymal transcription factors do not change in expression after OS2966 treatment of lung-seeking MDA-MB-231 breast cancer cells. Related to Figure 5D. Shown are qPCR results for six mesenchymal transcription factors in MDA-MB-231 lung lines after 48 hours of OS2966 treatment (n=3/group). *P<0.05; **P<0.01; ***P<0.001.

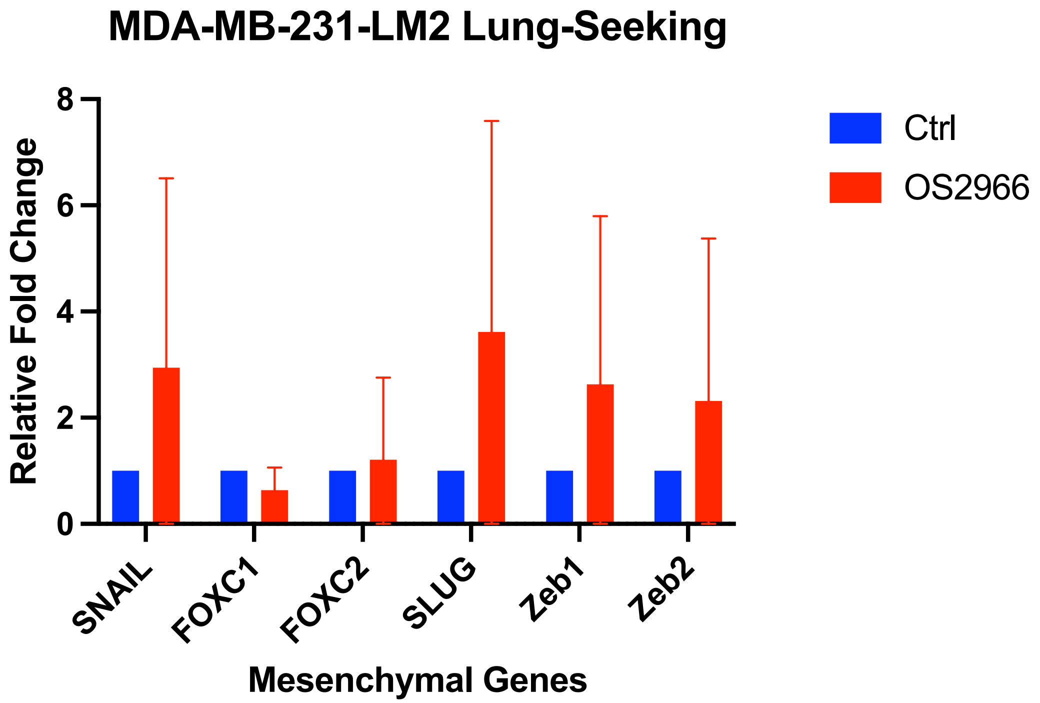

	Log2 fold cha	std error (log	Lower confid	Upper confid	Linear fold ch	Lower confid	Upper confid	P-value	BY.p.value	method	probe.ID
ZIC2-mRNA	-0.38	0.0503	-0.479	-0.282	0.768	0.718	0.823	0.00164	1	loglinear	NM_007129.2:1849
CASP3-mRNA	-0.13	0.0196	-0.168	-0.0913	0.914	0.89	0.939	0.00272	1	loglinear	NM_032991.2:685
VEGFA-mRNA	-0.636	0.122	-0.876	-0.397	0.643	0.545	0.76	0.00649	1	lm.nb	NM_001025366.1:1325
CDK2-mRNA	0.1	0.0205	0.0598	0.14	1.07	1.04	1.1	0.0082	1	loglinear	NM_001798.2:220
WNT7B-mRN	-0.744	0.14	-1.02	-0.471	0.597	0.494	0.722	0.0129	1	Wald	NM_058238.1:1535
FZD7-mRNA	-0.422	0.103	-0.624	-0.221	0.746	0.649	0.858	0.0148	1	loglinear	NM_003507.1:1890
CDKN1A-mR	-0.587	0.148	-0.877	-0.298	0.666	0.545	0.813	0.0165	1	lm.nb	NM_000389.2:1975
SPRY2-mRNA	-0.334	0.0883	-0.507	-0.16	0.794	0.704	0.895	0.0195	1	loglinear	NM_005842.2:85
EFNA5-mRNA	-0.367	0.0993	-0.562	-0.172	0.775	0.677	0.887	0.0209	1	loglinear	NM_001962.2:5035
HMGA2-mRN	-0.342	0.0999	-0.538	-0.147	0.789	0.689	0.903	0.0266	1	loglinear	NM_003484.1:328
RIN1-mRNA	-0.218	0.0651	-0.346	-0.0905	0.86	0.787	0.939	0.0285	1	loglinear	NM_004292.2:2572
PTEN-mRNA	-0.247	0.0776	-0.399	-0.0952	0.842	0.758	0.936	0.0333	1	loglinear	NM_000314.3:1675
SMAD9-mRN	-0.44	0.124	-0.683	-0.196	0.737	0.623	0.873	0.0386	1	Wald	NM_005905.2:1595
LAMC2-mRN	-0.496	0.166	-0.822	-0.171	0.709	0.566	0.889	0.0405	1	loglinear	NM_005562.2:2819
FGFR1-mRNA	-0.389	0.133	-0.649	-0.129	0.764	0.638	0.915	0.0428	1	loglinear	NM_015850.2:1335
LTBP1-mRNA	-0.448	0.156	-0.755	-0.142	0.733	0.593	0.906	0.0456	1	lm.nb	NM_000627.3:4124
NBN-mRNA	-0.144	0.0503	-0.242	-0.0453	0.905	0.845	0.969	0.0459	1	loglinear	NM_001024688.1:1105

Supplementary Table S1. Genes related to canonical cancer pathways that are upregulated by c-Met/b1 compelx formation.

	Undirected T	Directed Treatment: differential expression in AC vs. baseline of Ctrl
Cell Cycle - A	1.293	0.949
Chromatin M	1.14	0.988
DNA Damage	0.901	0.432
Driver Gene	1.072	0.862
Hedgehog	2.742	2.742
JAK-STAT	1.121	1.115
MAPK	1.224	1.203
Notch	1.151	1.151
PI3K	1.376	1.095
Ras	1.245	1.225
TGF-beta	1.218	1.139
Transcription	1.153	0.966
Wnt	1.474	1.432

Supplemental Table S2. Pathways activated by c-Met/β1 complex induction in breast cancer cells. Shown are the pathways activated when MDA-MB-231-iDimerize-c-Met-β1 cells were treated with AP21967 based on assessed in the NanoString nCounter platform using a 770 gene multiplex related to 13 cancer-associated canonical pathways

	Log2 fold cha	std error (log	Lower confid	Upper confid	Linear fold cl	Lower confid	Upper confid	P-value	BY.p.value	method	Gene.sets	probe.ID
PGK1-mRNA	0.296	0.0663	0.166	0.426	1.23	1.12	1.34	0.0111	1	loglinear	HIF1A Signali	NM_000291.2:1030
AKAP2-mRN/	-0.246	0.0566	-0.357	-0.135	0.843	0.781	0.911	0.0122	1	loglinear	Epithelial to I	NM_001004065.4:4956
FBN1-mRNA	-0.38	0.0877	-0.552	-0.208	0.768	0.682	0.866	0.0123	1	loglinear	Basement M	NM_000138.3:6420
ROCK1-mRN/	-0.141	0.0331	-0.205	-0.0758	0.907	0.867	0.949	0.0132	1	loglinear	Cell Adhesion	NM_005406.1:2660
TGFBR2-mRN	0.194	0.0483	0.0997	0.289	1.14	1.07	1.22	0.0158	1	loglinear	Cell Prolifera	NM_001024847.1:1760
BMPR2-mRN	-0.215	0.0563	-0.325	-0.105	0.862	0.798	0.93	0.0188	1	loglinear	Cell Prolifera	NM_001204.5:1875
ARAP2-mRN/	-0.212	0.0557	-0.321	-0.103	0.863	0.8	0.931	0.019	1	loglinear	Epithelial to I	NM_015230.2:4875
NAP1L3-mRN	0.855	0.19	0.482	1.23	1.81	1.4	2.34	0.0205	1	Wald	Epithelial to I	NM_004538.4:1070
VAMP8-mRN	0.421	0.121	0.183	0.659	1.34	1.14	1.58	0.0257	1	loglinear	Epithelial to I	NM_003761.3:260
AGR2-mRNA	1.46	0.424	0.625	2.29	2.74	1.54	4.88	0.0264	1	lm.nb	Epithelial to I	NM_006408.3:580
ENO2-mRNA	0.228	0.067	0.0962	0.359	1.17	1.07	1.28	0.0274	1	loglinear	HIF1A Signali	NM_001975.2:1855
COL7A1-mRN	-0.516	0.134	-0.779	-0.253	0.7	0.583	0.839	0.0311	1	Wald	Basement M	NM_000094.2:390
CDH11-mRN/	0.386	0.119	0.153	0.62	1.31	1.11	1.54	0.0315	1	loglinear	Cell Adhesion	NM_001797.2:1835
CXCR4-mRN/	-0.214	0.0665	-0.344	-0.084	0.862	0.788	0.943	0.0321	1	loglinear	Epithelial to I	NM_003467.2:1335
EPAS1-mRN/	0.474	0.158	0.165	0.783	1.39	1.12	1.72	0.0397	1	lm.nb	Angiogenesis	NM_001430.3:4246
RPS6KB2-mR	0.186	0.0624	0.0634	0.308	1.14	1.04	1.24	0.0409	1	loglinear	Cellular Grov	NM_003952.2:980
MED1-mRNA	-0.154	0.0544	-0.26	-0.0469	0.899	0.835	0.968	0.0477	1	loglinear	Angiogenesis	NM_004774.3:806

Supplemental Table S3. Genes whose expression is altered when β1 integrin cannot bind c-Met in breast cancer cells. Shown are the genes whose expression is altered when MDA-MB-231 breast cancer cells undergo CRISPRi knockdown of β1 integrin followed by lentiviral transduction of the β1D246A mutant vs. wild-type β1 integrin, as assessed in the NanoString nCounter platform using a 770 gene multiplex related to each step in the cancer progression process.

Supplementary Table S4. Primers used for qPCR. Shown are primers used for qPCR to assess expression of genes in breast cancer cells.

Gene Target	Forward	Reverse
STEM CELL PANEL		
c-Myc	5'- CAT CGT AAA CAC CAA CGT GC-3'	5'- CCG CGT TCA TGT CGT AAT AG- 3'
Klf4	5' –CAC CAT GCC GAT GTT CAT CGT AAA - 3'	5' –TTA GGC GAA GGT GGA GTT GT - 3'
Oct4	5' – CTT GCC TTG CTG CTC TAC CT – 3'	5' – CAC ACA GGA TGG CTT GAA GA – 3'
Sox2	5' – CAG CCA GAT GCA ATC AAT GC-3'	5-GCA CTG AGA TCT TCC TAT TGG TGA A-3'
Nanog	5'- GAC AAG CCA CAA GCT GAA CA-3'	5'- GAG CCC ACA ATG GGA GAGT A-3'
NANOSTRING VALIDATION		
Wnt7B	5'-AGC CAA CAT CAT CTG CAA CA-3'	5'-CTG GTA CTG GCA CTC GTT GA-3'
Fzd7	5'-CGC CTC TGT TCG TCT ACC TC-3'	5'-CCA TGA GCT TCT CCA GCT TC-3'
Zic2	5'-AAT CCC AAG AAG AGC TGC AA-3'	5'-ACA CTC CTC CCA GAA GCA GA-3'
VEGFA	5'-AGG CCA GCA CAT AGG AGA GA-3'	5'-TTT CTT GCG CTT TCG TTT TT-3'
CDKN1A	5'-GAC ACC ACT GGA GGG TGA CT-3'	5'-CCA CAT GGT CTT CCT CTG CT-3'
CDK2	5'-TTG TCA AGC TGC TGG ATG TC-3'	5'-TGA TGA GGG GAA GAG GAA TG-3'
CASP3	5'-TTT TTC AGA GGG GAT CGT TG-3'	5'-CGG CCT CCA CTG GTA TTT TA-3'
LAMC2	5'-GGC TGG TCT TAC TGG AGC AG-3'	5'-CAT CAG CCA GAA TCC CAT CT-3'
SMAD9	5'-CCA CAG AAG CCT CTG AGA CC-3'	5'-CCC AAC TCG GTT GTT CAG TT-3'
EFNA5	5'-ATG TGT GTG TTC AGC CAG GA-3'	5'-GGG CAG AAA ACA TCC AGG TA-3'
FGFR1	5'-CGA TGT GCA GAG CAT CAA CT-3'	5'-TGC TGG TTA CGC AAG CAT AG-3'
HMGA2	5'-CCT AAG AGA CCC AGG GGA AG-3'	5'-AAC TTG TTG TGG CCA TTT CC-3'
PTEN	5'-CGA CGG GAA GAC AAG TTC AT-3'	5'-AGG TTT CCT CTG GTC CTG GT-3'
RIN1	5'-CCC AGA CCT AGT CCA GCT CA-3'	5'-GGA GCT CCA GAA CTC AAT GC-3'
SPRY2	5'-ATC AGA GCC ATC CGA AAC AC-3'	5'-CAG ACC GTG GAG TCT CTC GT-3'

MESENCHYMAL TRANSCRIPTION FACTOR PANEL		
GSC	5'-TCT CAA CCA GCT GCA CTG TC	5'-GGC GGT TCT TAA ACC AGA CC-3'
FOXC1	5'-CAT CCG CCA CAA CCT CTC GCT-3'	5'-GTG CAG CCT GTC CTT CTC CTC C-3'
FOXC2	5'-GCC TAA GGA CCT GGT GAA GC-3'	5'-TTG ACG AAG CAC TCG TTG AG-3'
ZEB1	5'-GCA CCT GAA GAG GAC CAG AG-5'	5'-TGC ATC TGG TGT TCC ATT TT-3'
ZEB2	5' GAC AGA TCA GCA CCA AAT GC-3'	5'-GCT GAT GTG CGA ACT GTA GG-3'
SLUG	5'-CAC TAT GCC GCG CTC TTC-3'	5'-GGT CGT AGG GCT GCT GGA A-3'
SNAIL	5'-TGG TTG CTT CAA GGA CAC AT-3'	5'-GTT GCA GTG AGG GCA AGA A-3'
TWIST	5'-GGA GTC CGC AGT CTT ACG AG-3'	5'-TCT GGA GGA CCT GGT AGA GG-3'
ANALYZING TISSUES FOR MICROMETASTASES		
Luciferase	5’-GTG GTG TGC AGC GAG AAT AG-3’	5’-CGC TCG TTG TAG ATG TCG TTA G -3’
STANDARDS		
GAPDH	5'-CAA TGA CCC CTT CAT TGA CC-3'	5'-TTG ATT TTG GAG GGA TCT CG-3'
ACTB	5'-GAG CAC AGA GCC TCG CCT TT-3'	3'-ACA TGC CGG AGC CGT TGT C-3'

Supplemental Table 5: Antibodies used in this manuscript

Antigen	Species Source	Vendor	Catalog/Clone Number	Dilution Used	Fluorochrome Conjugation	Application
ITGB1	Mouse	Abcam	ab30394	1:250	N/A	Proximity Ligation Assay
ITGB1	Rabbit	Abcam	ab52971	1:40	N/A	Immunoprecipitation
ITGB1	Rabbit	Abcam	ab52971	1:1000	N/A	Western Blot
C-met	Rabbit	Abcam	ab51067	1:1000	N/A	Western Blot
C-met	Rabbit	Abcam	ab51067	1:250	N/A	Proximity Ligation Assay
Rabbit IgG	Donkey	Sigma-Aldrich	DUO92002	1:5	Duolink PLUS Probe	Proximity Ligation Assay
Mouse IgG	Donkey	Sigma-Aldrich	DUO92004	1:5	Duolink MINUS Probe	Proximity Ligation Assay
HA tag	Rabbit	Cell Signalling	3724	1:40	N/A	Immunoprecipitation
HA tag	Rabbit	Cell Signalling	3724	1:1000	N/A	Western Blot
Rabbit IgG	Goat	Cell Signalling	7074	1:8000	N/A	Western Blot
CD44	Mouse	Biolegend	338806	1:20	APC	Flow Cytometry
CD24	Mouse	Biolegend	311104	1:20	FITC	Flow Cytometry
